# miRNA/siRNA-directed pathway to produce noncoding piRNAs from endogenous protein-coding regions ensures *Drosophila* spermatogenesis

**DOI:** 10.1126/sciadv.adh0397

**Published:** 2023-07-19

**Authors:** Taichiro Iki, Shinichi Kawaguchi, Toshie Kai

**Affiliations:** Laboratory of Germline Biology, Graduate School of Frontier Biosciences, Osaka University, Yamadaoka1-3, Suita, Osaka, Japan.

## Abstract

PIWI-interacting RNA (piRNA) pathways control transposable elements (TEs) and endogenous genes, playing important roles in animal gamete formation. However, the underlying piRNA biogenesis mechanisms remain elusive. Here, we show that endogenous protein coding sequences (CDSs), which are normally used for translation, serve as origins of noncoding piRNA biogenesis in *Drosophila melanogaster* testes. The product, namely, CDS-piRNAs, formed silencing complexes with Aubergine (Aub) in germ cells. Proximity proteome and functional analyses show that CDS-piRNAs and cluster/TE-piRNAs are distinct species occupying Aub, the former loading selectively relies on chaperone Cyclophilin 40. Moreover, Argonaute 2 (Ago2) and Dicer-2 activities were found critical for CDS-piRNA production. We provide evidence that Ago2-bound short interfering RNAs (siRNAs) and microRNAs (miRNAs) specify precursors to be processed into piRNAs. We further demonstrate that Aub is crucial in spermatid differentiation, regulating chromatins through mRNA cleavage. Collectively, our data illustrate a unique strategy used by male germ line, expanding piRNA repertoire for silencing of endogenous genes during spermatogenesis.

## Teaser

A pathway where miRNA and siRNA produce piRNA expands the repertoire of small non-coding RNAs supporting sperm formation.

## INTRODUCTION

In eukaryotes, diverse biological processes are controlled by silencing mechanisms relying on 20– to 30–nucleotide (nt) small noncoding RNAs. Small RNAs (sRNAs) interact with Argonaute (Ago) family proteins inside RNA-induced silencing complexes (RISCs), acting as sequence-dependent guide for selecting silencing targets. PIWI-interacting RNAs (piRNAs) comprise a group of sRNAs forming RISCs with PIWI-clade Ago members and accumulating in animal gonads. piRNA-directed mechanisms are crucial in defending germline genomes against transposable elements (TEs) and controlling endogenous gene expression (*1*–*3*). Hence, piRNA pathway deficiencies can lead to failure in stem cell maintenance, functional gamete formation, and other processes. However, the diversity, biogenesis, and functions of piRNAs are not fully elucidated.

Selected transcripts can serve as precursors for piRNA production. Most of the precursors are defined intergenic regions called piRNA clusters, where truncated TEs can accumulate as remnant of past invasive activities (*4*–*6*). In *Drosophila melanogaster*, cluster regions are largely heterochromatinized and thus require a noncanonical mechanism for transcription initiation by RNA polymerase II. In germ cells, this relies on complexes containing heterochromatin protein 1 variant, [Rhino (Rhi)] and the cofactors (deadlock and cutoff) (*1*). Distinguished from canonical transcripts including mRNAs, cluster transcripts are exported via specialized machinery as piRNA precursors (*7*, *8*). Endonucleolytic cleavage, PIWI loading, and 3′ end trimming give rise to 23- to 29-nt mature forms of piRNAs (*9*–*12*). In addition to the above-mentioned pathway, germline piRNAs are amplified by ping-pong cycle mediated by cytoplasmic PIWI members, Aubergine (Aub) and Ago3 (*1*). Aub and Ago3 continue primary/trigger piRNA-directed reciprocal cleavages of sense and antisense TE sequences. Generated 3′ fragments are processed from 5′ end into secondary/responder piRNAs. Because target cleavage occurs at position bound by 10th to 11th nucleotides of trigger piRNAs, trigger and responder pairs hold 10-nt 5′-to-5′ complementarity. Similar to TE-related transcripts, a couple of endogenous protein-coding transcripts including *stellate* (*ste*), *vasa* (*vas*), and *pirate* (*pira*) can produce piRNAs via ping-pong, being targeted by trigger piRNAs derived from *su*(*ste*), *AT-Chx*, and *petrel* clusters, respectively (*13*–*15*). However, aside from these few examples, germ cells generally disfavor the entry of non-TE sequences including endogenous protein-coding transcripts to piRNA biogenesis, which ensures the fidelity of piRNA biogenesis for TE silencing (*9*, *16*).

Similar to *Drosophila*, murine fetal testes activate ping-pong and accumulate piRNAs containing TE-related repeat sequences (*17*). However, during neonatal period when spermatocytes and spermatids accumulate, cluster regions having fewer TE sequences are transcribed and processed into so-called pachytene piRNAs (*18*). Because of the low TE content, many pachytene piRNAs display unique sequences mapping only once to their origins in genome, thereby expanding the piRNA repertoire in testes. On the other hand, *Drosophila* major clusters are enriched with TE fragments and repetitive sequences. Nonetheless, endogenous protein-coding transcripts can serve as precursors of piRNA production and provide unique sequence repertoire (*19*, *20*). A defining characteristic of these genic piRNAs is their production from 3′ untranslated regions (3′UTRs). As exemplified by *traffic jam* (*tj*), a representative host gene, 3′UTR-piRNAs bind Piwi and exert gene regulatory effects in gonadal soma (*19*). Overall, however, TE-irrelevant unique sequence piRNAs have been poorly characterized in *Drosophila* germ cells.

Silencing pathways exhibit adaptation to testis-specific RNA metabolism. This is demonstrated with Ago2 that is binding to short interfering RNAs (siRNAs) and crucial in RNA-based immunity against foreign pathogens (*21*). However, in testes, a unique set of endogenous siRNAs (endo-siRNAs) are excised from hairpin transcripts by a ribonuclease (RNase) III enzyme Dicer-2 (Dcr2) and incorporated into Ago2 (*22*). Endo-siRNAs guide Ago2 and direct mRNA cleavage, thereby supporting late spermatogenesis (*22*–*24*). We recently reported a cochaperone associated with Hsp90 machinery, Cyclophilin 40 (Cyp40), is preferentially expressed in male germ line and essential for sperm formation (*25*). Ago2 binds testis-unique microRNAs (miRNAs) and endo-siRNAs, and Cyp40 increases the miRNA occupancy inside RISCs. Other functions of Cyp40 have remained elusive. In piRNA pathway, testicular Ago3 expression is restricted in spermatogonia (*26*). Hence, canonical ping-pong between Ago3 and Aub is supposed to be dampened in spermatocytes, while broadly expressed Aub alone can maintain the basal activity (*27*). Rhi shows testis-unique dynamic behaviors and protects early germ cells (*28*). piRNA pathways associated with later events in spermatogenesis have been uncertain.

In this study, we show that *Drosophila* male, but not female, germ cells activate a pathway to produce piRNAs displaying TE-irrelevant unique sequences derived from hundreds of endogenous protein-coding genes. These previously undocumented piRNAs can be distinguished from those known genic species by their origins in protein-coding sequences (CDSs) rather than in 3′UTRs and by their fates being loaded onto Aub but not Piwi. Furthermore, different from cluster/TE-piRNAs, neither Rhi-dependent cluster transcription nor Ago3-dependent ping-pong amplification is required for their production. Alternatively, their biogenesis relies on the activities of Cyp40 and Ago2. With regard to the latter, Ago2-bound siRNAs and miRNAs specify piRNA precursors and serve as triggers of secondary piRNA production. Generated piRNAs guide Aub for target mRNA cleavage, thereby directly involved in the control of endogenous gene expression for late spermatogenesis.

## RESULT

### *Drosophila* testes produce piRNAs from endogenous protein-coding sequences (CDSs)

**Fig. 1.**
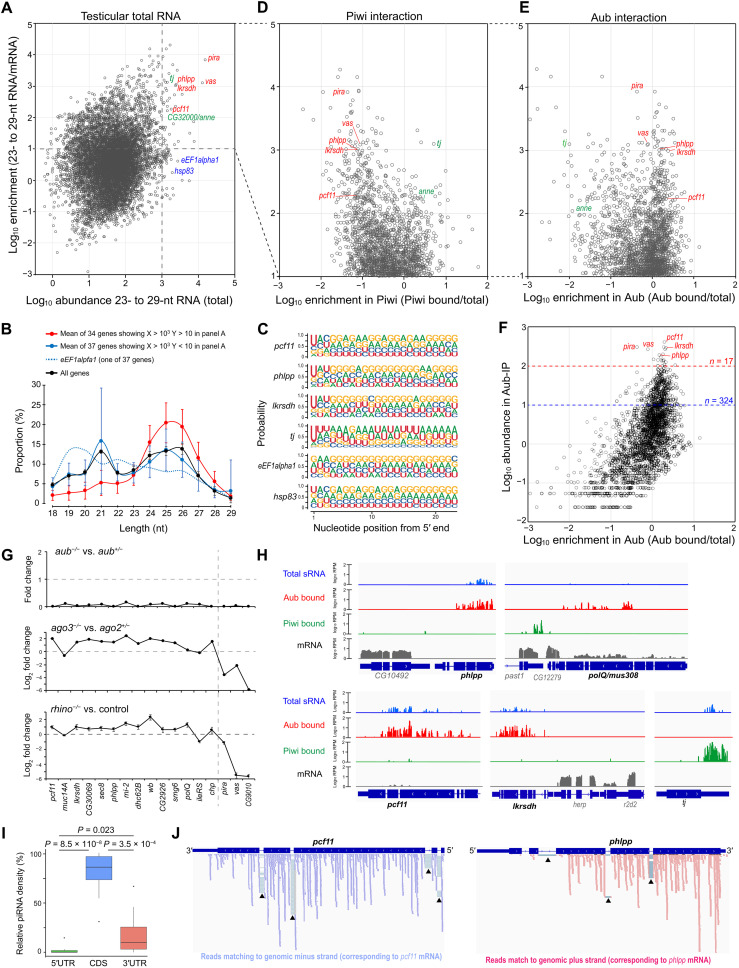
Testicular sRNA mapping to *D. melanogaster* gene exons. (**A**) Mapping of 23- to 29-nt RNAs accumulating in *D. melanogaster* testes to endogenous protein-coding genes. Abundance (TPM, transcripts per million) of 23- to 29-nt RNAs mapping to exons (5′UTR + CDS + 3′UTR) was obtained for individual genes. Mean of 6 data was shown in *x* axis. Mean mRNA abundance (TPM) was obtained from two data, and enrichment of 23- to 29-nt RNAs relative to mRNAs (TPM/TPM) was shown in *y* axis. (**B**) Size distribution of 18- to 29-nt exon-mapping RNAs. (**C**) Nucleotide probability of 23- to 29-nt exon-mapping RNAs. (**D** and **E**) Binding of 23- to 29-nt RNAs to Piwi or Aub. Abundance (TPM) of 23- to 29-nt RNAs in Piwi- or Aub-bound fraction was given as mean of Piwi-immunoprecipitation (IP) and GFP-Piwi-IP data or as mean of Aub-IP and GFP-Aub-IP data. Enrichment (Piwi bound/total or Aub bound/total) was shown in *x* axis. Genes enriched with 23- to 29-nt RNAs relative to mRNAs (TPM/TPM > 10, *n* = 2449) were analyzed. (**F**) Genes producing piRNAs most abundantly for Aub. Abundance [mean RPM (read per million)] of Aub-IP and GFP-Aub-IP) was shown in *y* axis. Seventeen or 324 genes (RPM >100 or > 10) were grouped and analyzed later. (**G**) Loss of *aub*, *ago3*, or *rhi* effect on exon-mapping piRNAs. Seventeen genes (Aub-IP RPM >100) were analyzed. (**H**) Origins of piRNAs within gene exons. Bedgraph shows 23- to 29-nt RNAs in testis total RNAs (blue), Aub-bound piRNAs (red), Piwi-bound piRNAs (green), and mRNA transcriptome (gray). Gene model shows CDS (bold line) and UTRs (thin line). (**I**) Density of mapping piRNAs compared between 5′UTR, CDS, and 3′UTR. *pira*, *vas*, *CG9010* accumulating *rhi*- and *ago3*-dependent piRNAs were excluded from 17 genes. Remaining 14 genes were analyzed in box plot. *P* values (two-tailed paired *t* test) were indicated. (**J**) Strand orientation of Aub-bound piRNAs mapping to *pcf11* and *phlpp*. Arrowhead highlights exon-exon junction reads.

To deepen the understanding of piRNA pathways underlying spermatogenesis, we first characterized the genomic origins of piRNAs accumulating in testes of *D. melanogaster*. Analyses of deep sequencing (deep-seq) data, prepared in house and available in public (table S1), revealed that 23- to 29-nt piRNA-like reads accumulate to the exons of endogenous protein-coding genes to different degrees (Fig. 1A and fig. S1A). However, total RNAs contain silencing-irrelevant short fragments such as mRNA decay intermediates, leading to false-positive piRNA identification. Considering decay fragment accumulation could correlate with mRNA level, we plotted the abundance of 23- to 29-nt RNAs relative to mRNAs in individual genes and excluded those giving lower values from further analyses (*y*-axis value < 10; Fig. 1A). One of these excluded genes, *eEF1alpha1*, indeed displayed a broad fragment distribution ranging from 18 to 29 nt, possibly reflecting its degradome (Fig. 1B, dotted line). In contrast, the size profile of reads mapping to remained genes showed a clear peak at 24 to 26 nt, indicative of piRNAs (Fig. 1B, red line). In addition to size, these reads displayed uridine (U) enrichment at 5′ nucleotide, a feature of piRNAs bound to Piwi or Aub (Fig. 1C).

Of those genes enriched with 23- to 29-nt RNAs (*y*-axis value > 10, *n* = 2449; Fig. 1A), *traffic jam* (*tj*) and *CG32000/anne* are known to produce piRNAs interacting with Piwi (*19*). However, others including *phlpp*, *lkrsdh*, and *pcf11* have not been linked to piRNA production thus far. Comparison of 23- to 29-nt RNA abundance between total and Piwi-bound pools showed that testicular Piwi interacts with piRNAs derived from *tj* and *anne* (Fig. 1D). In marked contrast, 23- to 29-nt RNAs from many other genes did not exhibit affinity to Piwi but to Aub alternatively (Fig. 1, D to F). First, U bias and 24- to 26-nt peak were confirmed for Aub-bound reads mapped on individual genes [17 of 17 read per million (RPM) > 100 genes in fig. S1, B and C and 318 of 324 RPM > 10 genes in table S2]. Moreover, these piRNA-like products were nearly depleted in total RNAs of *aub* mutant testes (Fig. 1G). Together, these data indicate endogenous protein-coding gene exons produce bona fide piRNAs interacting with Aub in testicular germ cells.

Of 17 genes producing piRNAs most abundantly, *vas* and *pirate* (*pira*) are known to be targeted by piRNAs from *AT-ChX* and *petrel* clusters, respectively; secondary piRNAs can be generated from their cleaved mRNAs, such as TEs and cluster transcripts (*14*, *15*). Consistently, the levels of *vas-* and *pira*-derived piRNAs showed severe decrease in testes lacking *rhi* or *ago3,* which is crucial for cluster transcription or ping-pong amplification, respectively (Fig. 1G). In sharp contrast, piRNAs from other 14 genes excluding *CG9010* did not decrease their abundance, implying a distinct mechanism underlying their biogenesis. We further found piRNAs from these 14 members were predominantly mapped to protein-coding regions, a feature differed from known genic species mainly derived from 3′UTRs (Fig. 1, H and I). Considering these characteristics, we hereafter refer these genic piRNAs, representatively produced by aforementioned 14 genes, as endogenous CDS-derived piRNAs. CDS-piRNAs showed a near uniform sense-strand orientation corresponding to mRNAs, with an exception of *muc14A* dominated with antisense reads (fig. S1, D and E). In addition, CDS-piRNAs were rarely derived from introns, and a fraction of reads contained exon-exon junction sequences (Fig. 1J). These features suggest that spliced, sense-strand transcripts including mRNAs can serve as the precursors.

We examined whether CDS-piRNA production is regulated during germline development. Reanalysis of sRNAs present in testes lacking germline differentiation factors (*27*) showed that CDS-piRNAs are barely detectable in early spermatogonial cells but can be seen in primary spermatocytes (fig. S1F). These results suggest that CDS-piRNA production is induced as germ cells differentiate from spermatogonia to spermatocytes, a pattern opposed to cluster/TE-piRNA production.

### CDS-piRNAs are preferentially produced in testes compared to ovaries

**Fig. 2.**
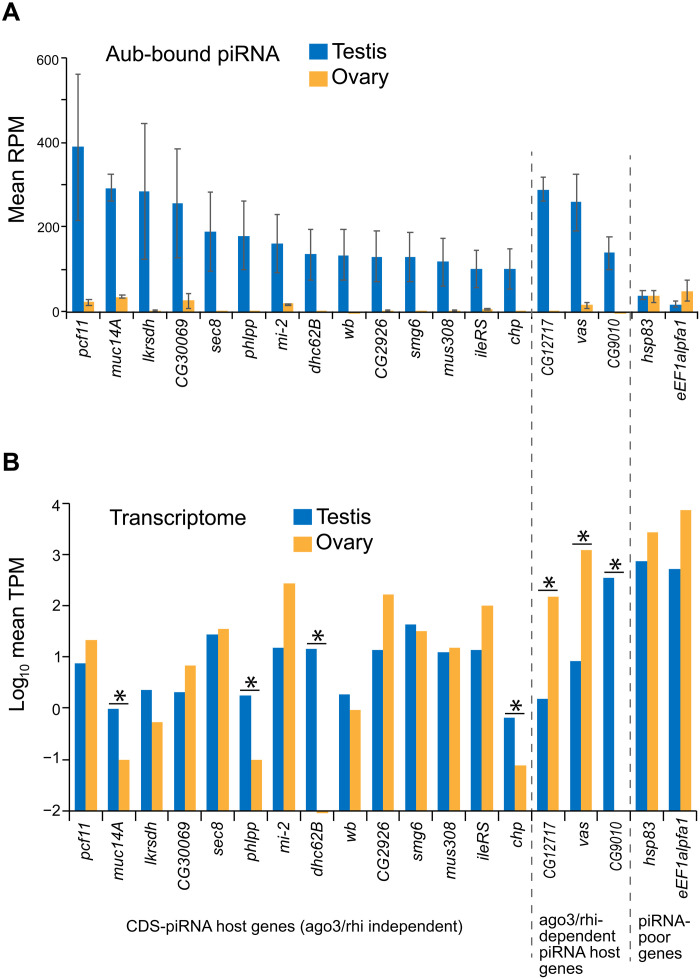
CDS-piRNAs and cognate mRNAs present in testes and ovaries. (**A**) CDS-piRNA abundance (RPM) inside Aub-RISCs was compared between testes and ovaries. Mean ± SD of two data was shown. Indicated genes include 17 representative piRNA producers in testes, together with two piRNA poor references (*hsp83* and *eEF1alpha1*). (**B**) Transcript abundance (TPM) of indicated genes was compared between testes and ovaries. Mean of four data was shown. Asterisk, FDR < 0.0001.

To examine whether ovaries can produce CDS-piRNAs same as testes, we mined the publicly available data of total sRNAs and Aub-bound piRNAs in ovaries (table S1) and compared with testicular datasets (fig. S2A). Curiously, ovarian Aub did not accumulate CDS-piRNAs mapping to genes identified in testes (Fig. 2A). Moreover, de novo screening of genes hosting piRNAs using ovarian datasets listed up much fewer candidates compared to those in testes (fig. S2, B and C). On the other hand, by genome-wide profiling, we did not observe significant difference in the proportion of exon mappers between testicular and ovarian data, possibly due to non-piRNA fragment contamination (fig. S2D). Overall, these results suggest that CDS-piRNAs are preferentially produced in testes rather than ovaries.

Contrary to CDS-piRNAs, the transcript levels of many (10 of 14) host genes were comparable between ovaries and testes (Fig. 2B). Therefore, lessor accumulation of Aub-bound CDS-piRNAs in ovaries would not be solely attributable to the fewer amount of precursor transcripts. Rather, these data imply testicular germ cells express factors that can act on the selected protein-coding transcripts and enable their processing into piRNAs.

### Aub is physically linked to Cyp40, an Hsp90 cochaperone specialized for spermatogenesis

**Fig. 3.**
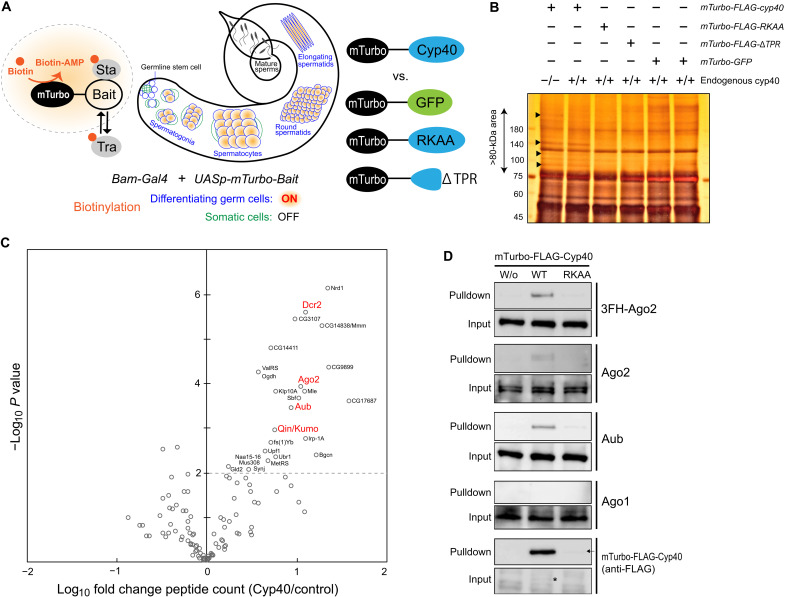
Cyp40 client screening in testicular germ cells using TurboID. (**A**) Principles of germline BioID/TurboID developed in this study. Using free biotins, an engineered biotinylation enzyme called mTurbo generates diffuse pattern of biotin–adenosine 5′-monophosphate (AMP), which adds biotins to lysine residues of proteins close to the selected bait. Not only stable (Sta) but also transient (Tra) interactors such as chaperone clients can be biotin-labeled. mTurbo was fused to bait proteins including Cyp40, nonfunctional Cyp40 derivatives (RKAA or ∆TPR), or GFP. RKAA and ∆TPR lack affinity to Hsp90. (**B**) Silver staining of proteins purified with streptavidin from testes. Cyp40-dependent signals (arrowheads) were enriched in >~80-kDa area from which peptides were extracted and analyzed by MS. (**C**) Volcano plot summarizing MS data. Spectrum counts of proteins purified from testes expressing mTurbo-FLAG-Cyp40 (two data; in the presence or absence of endogenous *cyp40*) were compared with those in control conditions (four data; single replicate of mTurbo-FLAG-Cyp40^RKAA^ and mTurbo-FLAG-Cyp40^∆TPR^, duplicate of mTurbo-GFP). *x* axis, enrichment of spectrum counts (Cyp40/control); *y* axis, *p* (two-tailed unpaired *t* test). Names of significantly (*P* < 0.01) enriched proteins were indicated. (**D**) Immunoblotting of Ago proteins and mTurbo-FLAG-Cyp40 present in testes (input) and purified with streptavidin (pulldown). For Ago2, both 3FLAG-hemagglutinin–tagged (3FH-)Ago2 expressed under native promoter activity and endogenous Ago2 were analyzed. Notably, mTurbo-FLAG-Cyp40 proteins were barely detectable in input (indicated by asterisk) but strongly enriched by streptavidin pulldown, indicating efficient self biotinylation. Steady-state level of mTurbo-FLAG-Cyp40^RKAA^ was much lower and only visible in pulldown fraction (arrow). WT, wild type.

What testicular factors can activate CDS-piRNA production and Aub loading? sRNA loading step in RISC formation relies on Hsp70/Hsp90 chaperone machineries (*29*–*32*). In *Drosophila*, an Hsp90 cochaperone Cyp40 is expressed in testicular germ cells and essential for sperm formation; however, its substrates/clients are elusive besides Ago2 (*25*). Hence, we performed Cyp40 client screening based on proximity-dependent biotin identification (BioID/TurboID) (*33*, *34*), which could be advanced in identifying transient chaperone-client interactions compared to conventional immunoprecipitation analyses (Fig. 3A). Cyp40 fused to an engineered biotin ligase termed mini Turbo (mTurbo) was expressed in germ cells using UASp-Gal4 system (Fig. 3A) (*35*). mTurbo-Cyp40 rescued the defective spermatid differentiation and sperm storage in *cyp40*-null testes, confirming the functionality of fusion construct (fig. S3A). In denaturing gel electrophoresis, potentially biotinylated proteins were detected as distinct bands when Cyp40, but not green fluorescent protein (GFP) or nonfunctional Cyp40 variant (RKAA or ∆TPR) was used as bait (Fig. 3B) (*25*).

The screening identified dozens of proteins in the physical proximity of Cyp40. Consistent with our previous findings (*25*), these included Ago2 and, in addition, its essential partner Dcr2 (Fig. 3C and fig. S3B). Intriguingly, Aub was identified as one of proximity factors and candidate clients of Cyp40. Moreover, our profiling listed up Qin/Kumo, a Tudor domain protein associated with Aub-RISC formation (*36*, *37*). Contrastively, no significant signal enrichment was seen for other Ago members. Immunoblotting using respective Ago antibodies confirmed the mass spectrometry (MS) data (Fig. 3D and fig. S3C). Notably, Cyp40-based TurboID did not list up Hsp90, the partner of Cyp40 in chaperone machinery and thus an expected biotinylation target. This perhaps implicates the relative distance between mTurbo and Hsp90 in complexes or other restrictions of used strategy. Nevertheless, the unbiased screening revealed the selective link of Cyp40 to Aub and Ago2 among Ago family members.

### Aub loading with CDS-piRNAs is selectively assisted by Cyp40

**Fig. 4.**
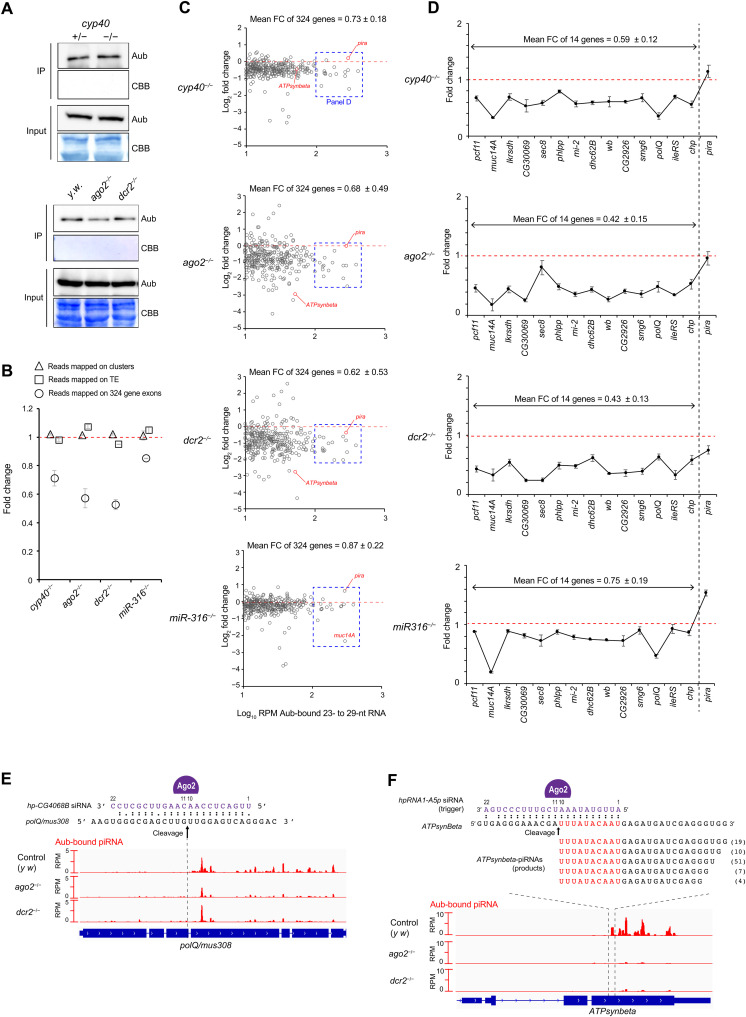
Molecular requirement for CDS-piRNA biogenesis. (**A**) Immunoblotting of endogenous Aub proteins present in testes (Input) and immunopurified with anti-Aub antibodies for deep-seq (IP). *cyp40^KO/Df^* null mutant (−/−) was compared to the sibling *cyp40*^*KO*/*6*^ heterozygous control (+/−). *ago2^454/Df^* and *dcr2^L811fsX/811fsX^* null mutants (−/−) were compared to *y w* control testes. Coomassie brilliant blue (CBB) staining serves as protein loading control. (**B**) Effect of *cyp40*, *ago2*, *dcr2*, or *miR-316* loss on piRNA abundance (RPM) inside Aub-RISCs. Aub-bound piRNAs were grouped as cluster mappers, TE mappers, or exon mappers. Mismatches were allowed when mapping to clusters or TEs. For exon mappers, 324 genes were analyzed to enrich CDS-piRNAs (Aub-IP RPM > 10; see Fig. 1F). Fold changes were given by *cyp40* mutant/heterozygous, *ago2* mutant/*y w*, and *dcr2* mutant/*y w* comparisons, respectively. Mean ± SD of two dataset was shown. (**C** and **D**) Effect of *cyp40*, *ago2*, *dcr2*, or *miR-316* loss on exon mappers in individual genes. piRNA-enriched 324 genes (C) and 14 (+1 = *pira*) genes (D) were analyzed. *Pira* serves as a reference producing piRNAs in a *ago3*/*rhi*-dependent manner, unlike CDS-piRNAs. (**E** and **F**) siRNA target sites and piRNA origins on *mus308/polQ* and *ATPsynbeta*. In *ATPsynbeta,* targeting siRNAs and produced piRNAs exhibit 5′-to-5′ 10-nt complementary overlap. Numbers next to piRNA sequences indicate read counts in merged Aub-IP data (*y w*).

Above results prompted us to explore a possibility that Cyp40 is involved in Aub-dependent piRNA pathways in testes. Given chaperone functions are associated with RISC formation, Cyp40 could affect piRNAs inside Aub-RISCs. To examine this possibility, Aub-bound piRNAs in *cyp40* null testes were analyzed by deep-seq and compared with those of heterozygous siblings. Aub protein level was comparable between those samples (Fig. 4A and fig. S4A), and, accordingly, the abundance of piRNAs mapping to cluster or TE sequences were largely unaffected (Fig. 4B and fig. S4B). In sharp contrast to cluster/TE-piRNAs, however, exon mappers enriched with CDS-piRNAs (exons of 324 genes; Fig. 1F) were reduced in *cyp40* mutant testes [fold change (FC) = 0.71 ± 0.05; Fig. 4B]. Individual analyses of 324 genes confirmed the general trend of mapping reads reduction (FC = 0.73 ± 0.18; Fig. 4C and table S3). Of those, *pira* was one of exceptions; similar to cluster/TE-piRNAs, its piRNA level was unaffected (Fig. 4D). In sum, bona fide CDS-piRNAs derived from 14 genes (Fig. 1G) exhibited a severe decrease in abundance upon loss of *cyp40* from testes (FC = 0.59 ± 0.12; Fig. 4D). These results suggest that Cyp40 can distinguish CDS-piRNAs from cluster/TE-piRNAs and selectively supports the former accumulation in Aub. The uncovered *cyp40* dependency, together with the *rhi/ago3*-independency (Fig. 1G), supports the idea that CDS-piRNA production is distinct from that of cluster/TE-piRNAs.

### CDS-piRNA production relies on Ago2-Dcr2 activities

CDS-piRNAs often exhibit mapping preference within the protein-coding regions, as being exemplified by DNA polymerase theta (*polQ*)/*mus308* and ATP synthase beta (*ATPsynbeta*; one of 324 genes). Their transcripts are known targets of siRNAs derived from *CG4068* and *hpRNA1*, respectively, and cleaved by Ago2 (Fig. 4, E and F) (*22*, *23*, *38*). We found, for both genes, that piRNAs were near exclusively mapped downstream of individual cleavage sites. Particularly for *ATPsynbeta*, a fraction of piRNAs held 5′ -to-5′ 10-nt complementary overlap with targeting siRNAs, suggesting that the 3′ cleavage fragments can be directly transferred to Aub for processing (Fig. 4F).

These observations imply that Ago2 and binding sRNAs can act as triggers for the production of at least some of CDS-piRNAs. To examine this possibility, Aub-bound piRNAs in *ago2*- or *dcr2*-null mutants were analyzed by deep-seq. Similar to *cyp40* analyses, loss of *ago2* or *dcr2* did not alter the abundance of Aub proteins and cluster/TE-piRNAs (Fig. 4, A and B, and fig. S4B) but contrastively results in severe reduction of CDS-piRNA–enriched exon mappers (FC = 0.57 ± 0.06 and 0.53 ± 0.04; Fig. 4B). Individual genes exhibited more variable effects (FC = 0.68 ± 0.49 and 0.62 ± 0.53; Fig. 4C), but, nonetheless, there was correlation between *ago2*- and *dcr2*-deficient conditions (*r* = 0.48; fig. S4C). To this end, genuine CDS-piRNAs from 14 genes showed most severe decrease (FC = 0.42 ± 0.15 and 0.43 ± 0.13; Fig. 4D).

Our data highlight a set of genes including *ATPsynbeta* whose piRNAs were extremely sensitive to the loss of Ago2-Dcr2 pathway; however, these cases were rare (Fig. 4, C and F, and fig. S4C). Consistently, our screening identified only few CDS-piRNAs exhibiting 10-nt overlap with Ago2-bound si/miRNAs (fig. S4D and table S4). Hence, unlike *ATPsynbeta*, coupling between Ago2 cleavage and Aub loading might be infrequent. Overall, our data suggest that Ago2-Dcr2 pathway is important, if not essential, for the global CDS-piRNA production.

We further investigated how Ago2 activates CDS-piRNA production by examining if Ago2-bound miRNAs are involved. We previously reported that *miR-316* binds its 3p strand to testicular Ago2 in a Cyp40-dependent manner and functions in spermatogenesis (*25*). The effect of *miR-316* loss on global CDS-piRNA levels was the weakest among the analyzed mutants (Fig. 4, B and C). Nonetheless, a moderate correlation was observed between *miR-316* and *cyp40* (*r* = 0.59; fig. S4C), suggesting their functional interaction in CDS-piRNA production. Conspicuously, gene-by-gene analysis revealed that *muc14A* exhibits a severe decrease in the piRNA level (Fig. 4, C and D, and fig. S4F). Notably, *muc14A* is unique by containing ~300-nt repeats inside the coding region (fig. S4E). Accordingly, its atypical antisense-enriched CDS-piRNAs (fig. S1, D and E) were solely derived from *muc14A* but contained intragenic multimappers forming a hotspot (fig. S4F). To support the direct involvement of *miR-316-3p* in *muc14A*-piRNA production, a total of 18 target sites were identified and accumulated in *muc14A* antisense sequence (no site in sense sequence), although these sites would be largely cleavage incompetent due to the central mismatches (fig. S4F). Nonetheless, predicted target sites located nonrandomly and surrounded the piRNA hotspot, fitting to the idea that *miR-316-3p*–directed Ago2 can specify piRNA precursor region within target transcripts. Together, these data suggest that Ago2 uses both siRNAs and miRNAs for CDS-piRNA production.

Multiple piRNAs can be generated from a single precursor transcript by phasing mechanism relying on Zucchini (Zuc)–mediated cleavage (*9*, *10*). We found that CDS-piRNAs derived from genes including *muc14A* show phasing signatures (fig. S4G). This result suggests that si/miRNAs can direct Zuc-dependent, phased CDS-piRNA biogenesis.

### *aub* is crucial for spermatid differentiation and male fertility

**Fig. 5.**
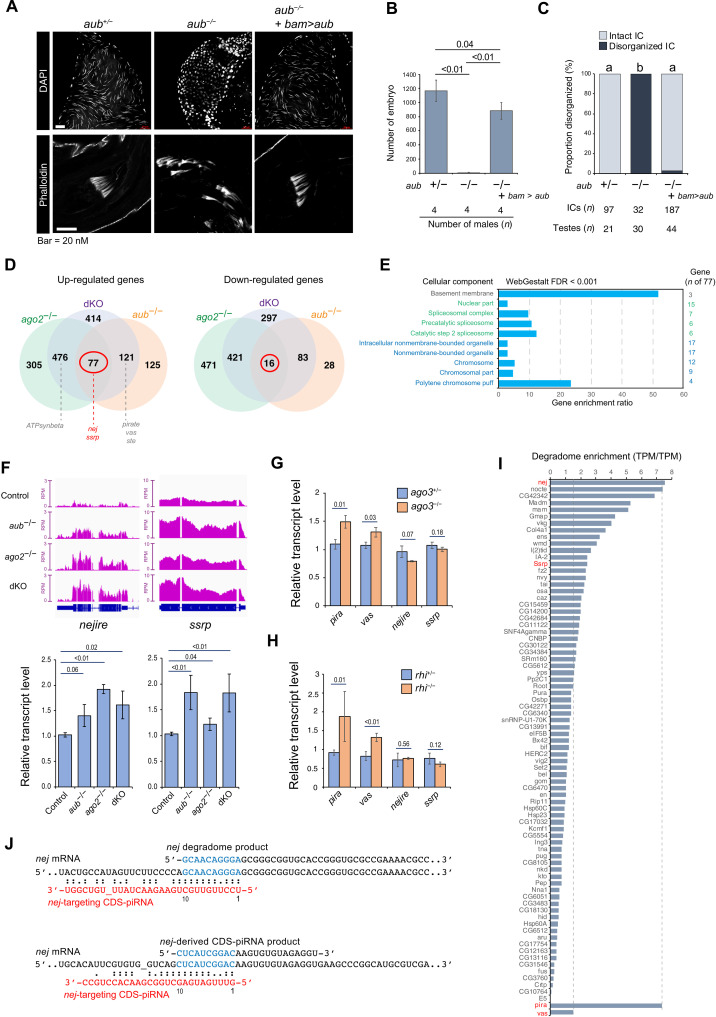
Functions of Aub, Ago2, and CDS-piRNAs in late spermatogenesis. (**A**) Defective spermatogenesis in testes lacking *aub* (−/−; *aub^N11/HN2^*) and the rescue by *aub* transgene (*bam-Gal4>UASp-mTurbo-FLAG-aub*). *aub* heterozygous siblings (+/−) serve as control. Sperm nuclei stored in seminal vesicles [4′,6-diamidino-2-phenylindole (DAPI)]. ICs in elongating spermatids (phalloidin). (**B**) Male fertility test. Mean ± SD was shown. *P* values (two-tailed unpaired *t* test) were indicated. (**C**) Disorganized IC counting. IC containing more than five visibly-dissociated actin corns was defined as “disorganized.” Differential characters indicate statistically significant difference (*P* < 0.01) in Tukey’s test. Number of analyzed testes and counted ICs was indicated. (**D**) Venn diagram highlights genes regulated by both *aub* and *ago2*. Transcriptome of *aub^N11/HN2^* mutant (*aub^−/−^ ago2^+/−^*), *ago2^454/Df^* mutant (*aub^+/−^ ago2^−/−^*), or dKO (*aub^−/−^ ago2*^*−/*−^) was compared to that of sibling control (*aub^+/−^ ago2^+/−^*). Biological duplicate data were analyzed, and the number of differentially expressed genes was indicated (FDR < 0.0001). (**E**) Gene Ontology (cellular components) analyses of 77 genes overexpressed in all mutant conditions. (**F**) Deep-seq profile (bedgraph) and qPCR measurement (bar graph) of *nej* and *ssrp* transcripts. Mean ± SD was shown. *P* values given by two-tailed unpaired *t* test were indicated. Biological replicates: 3 (*nej*) and 4 (*ssrp*). (**G** and **H**) qPCR measurement of gene transcripts in *ago3^t2/t3^* (−/−) or *rhi^02086/Df^* (−/−) compared to respective heterozygous sibling controls (+/−). Mean ± SD was shown. *P* values given by two-tailed unpaired *t* test were indicated. Biological replicates: 3 (*ago3*) and 4 (*rhi*). (**I**) Enrichment of degradome products relative to polyadenylated transcripts (TPM/TPM). Genes overexpressed in *aub* and *ago2* mutants (*n* = 77) were analyzed. *pira* and *vas* serve as reference target of piRNAs for cleavage. (**J**) Signatures of CDS-piRNA–directed *nej* mRNA cleavage. 5′-to-5′ 10-nt complementary overlaps between targeting CDS-piRNAs and degradome fragments (top) and between targeting CDS-piRNAs and produced *nej-*derived piRNAs (bottom).

As a step toward understanding the biological roles of CDS-piRNA pathway, we examined if and how *aub* is required for spermatogenesis. A loss-of-function allele, *aub^N11/HN2^*, contained much fewer sperms in seminal vesicles compared to the heterozygous siblings, and the males were nearly sterile (Fig. 5, A and B, and fig. S5A). Close examination of spermatogenesis revealed that, although spermatogonia and spermatocytes were largely unaffected (fig. S5B), individualization complexes (ICs) formed by spermatids were severely disorganized (Fig. 5, A and C). Those defects were largely recovered by introduced *aub* transgene, indicating that the loss of *aub* is causal for the phenotypes. Similar to *aub*, *ago2* is important for CDS-piRNA production (Fig. 4) and known to play key roles in late spermatogenesis (fig. S5, B and C) (*22*). In addition, double knockout (dKO) of *aub* and *ago2* did not exhibit any discernible defects in spermatogonia and spermatocytes, similar to single KOs (fig. S5, B and C). These results suggest that CDS-piRNA pathway involving both Aub and Ago2 can support late spermatogenesis.

### CDS-piRNAs can exert regulatory effect on endogenous gene expression

CDS-piRNAs are enriched with TE-unrelated sequences, and their impacts on TEs are suspicious. Considering this and to examine the gene-regulatory functions of CDS-piRNAs, we profiled the transcriptome of testes deficient for CDS-piRNA production. Testes lacking *aub* (*aub^−/−^ ago2^+/−^*), *ago2* (*aub^+/−^ ago2^−/−^*), or both (dKO; *aub^−/−^ ago2^−/−^*) were obtained from progenies derived from the same parents, together with those of sibling heterozygous controls. Genes controlled by CDS-piRNA pathway could be misregulated in all mutant conditions. Consistent with previous studies, *ATPsynbeta*, a direct target of *hpRNA1*-derived siRNAs, was identified as one of overexpressed genes in *ago2* mutant and dKO testes, but not in *aub* mutants (Fig. 5D and fig. S5D) (*22*). Similar to *ATPsynbeta*, other genes producing CDS-piRNAs increased their transcript levels (fig. S5E), further supporting a role for Ago2 as processing trigger (Fig. 4). On the other hand, cluster-piRNA targets including *ste, pira*, and *vas,* were derepressed in *aub* mutant and dKO testes, but not in *ago2* mutants.

To explore genes under the control of CDS-piRNA pathway, we then listed up genes mistegulated both in *aub* and *ago2* mutants. The list contained 77 up-regulated and 16 down-regulated genes [false discovery rate (FDR) < 0.0001; Fig. 5D and table S5], and all were misregulated in the same way in dKO. Gene Ontology analyses indicated that overexpressed genes were significantly enriched with those functions associated with chromosome, splicing, and basement membrane (FDR < 0.001; Fig. 5E). The chromosome category included *nejire* (*nej*)/*CBP* and structure-specific recognition protein (*ssrp*), for which quantitative polymerase chain reaction (PCR) was performed to confirm the transcriptome output (Fig. 5F). Notably, *nej* encoding histone acetyltransferase is involved in spermatid differentiation and male fertility (*39*). Loss of *rhi* or *ago3* did not affect *nej* and *ssrp* levels; thus, their regulation by cluster/TE-piRNA pathway is unlikely (Fig. 5, G and H). CDS-piRNAs may recognize and direct their transcript cleavage. Degradome profiling revealed both genes are enriched with 5′ monophosphate fragments that can be made by endonucleolytic cleavage (Fig. 5I). An extreme case is *nej*, whose fragment enrichment was comparable to that of *pira*, tightly regulated by cluster-piRNAs via cleavage (*15*). To support the idea that *nej* cleavage is directed by CDS-piRNAs, 13 of 223 groups of degradome fragments were paired with CDS-piRNAs through 5′-to-5′ 10-nt complementary overlap (Fig. 5J and fig. S5F). Moreover, *nej* itself is producing piRNAs as one of the 324 genes (Fig. 1F), and 38 of 449 groups of *nej-*derived piRNAs were paired with CDS-piRNAs from other genes, implying that cleavage by CDS-piRNAs can trigger piRNA production from *nej* (Fig. 5J and fig. S5F). Together, these results suggest that CDS-piRNAs can directly regulate endogenous genes via mRNA cleavage.

Nonetheless, the transcript levels of *nej* and other genes can be affected by many different factors in *aub* and *ago2* mutant testes. These would include the independent activities of Aub and Ago2 beside their cooperation through CDS-piRNAs. Further investigations are needed to understand the direct silencing effects induced by CDS-piRNAs during spermatogenesis.

### Loss of Aub and interacting piRNAs causes overaccumulation of histone H4 acetylations and failure of histone-to-protamine transition

**Fig. 6.**
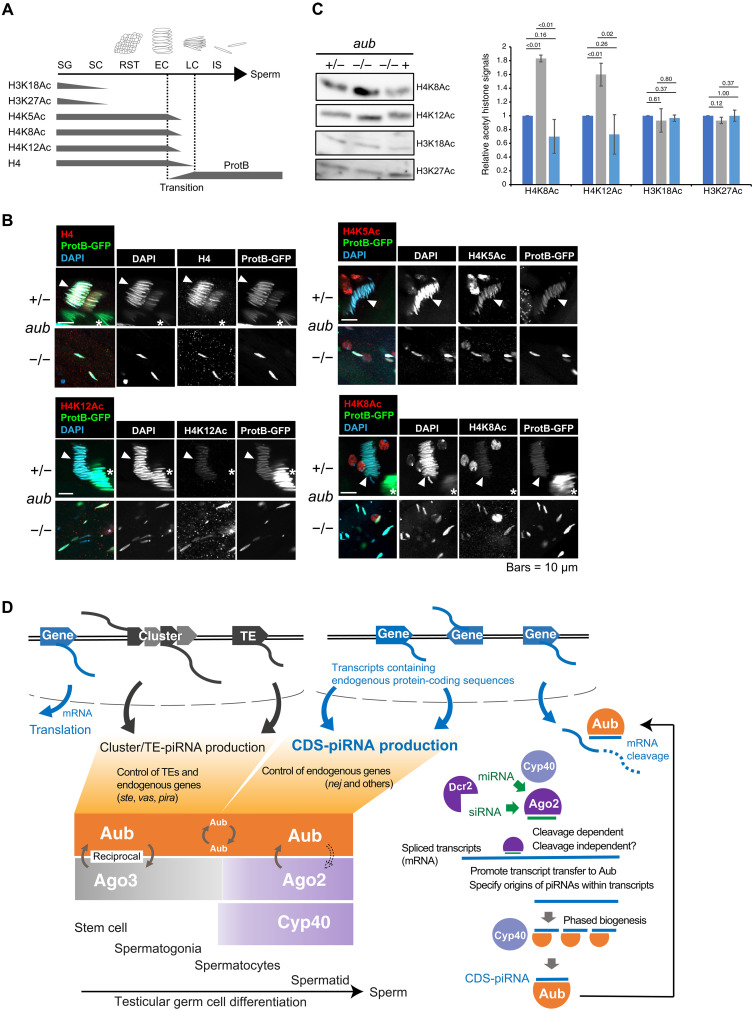
A role of Aub in histone-to-protamine transition and a model of piRNA biogenesis in testicular germ cells. (**A**) Summarized histone H3 and H4 acetylation patterns during spermatogenesis. SG, spermatogonia; SC, spermatocytes; RST, round spermatids; EC, early canoe stage spermatids; LC, late canoe stage spermatids; IS, individualizing spermatids. (**B**) Spermatid nuclei in testes of *aub* heterozygous control (+/−) or *aub^N11/HN2^* null mutants (−/−). Arrowhead denotes nuclei under transition containing both H4 and ProtB signals. Asterisk denotes nuclei after transition containing only ProtB signals. (**C**) Immunoblotting of acetyl histone H3 and H4 extracted from testes of *aub* heterozygous control (+/−) or *aub^N11/HN2^*-null mutants in the absence (−/−) or presence (−/− +) of *aub* transgene expression (*bam-Gal4>UASp-mTurbo-FLAG-aub*). Bar graph shows the mean ± SD of immunoblot signal intensities from biological triplicate data. *P* values given by two-tailed unpaired *t* test were indicated. (**D**) Model of piRNA biogenesis and functions in *Drosophila* male germ line. In spermatogonia and early spermatocytes, piRNAs are actively produced from cluster transcripts and TE mRNAs via ping-pong amplification. When ping-pong activity is reduced in spermatocytes, Aub can load fragments containing endogenous protein-coding sequences and form RISCs with CDS-piRNAs. Cyo40, Ago2, and binding mi/siRNAs assist Aub to form CDS-piRNA-RISCs. Control of genes through CDS-piRNA–directed mRNA cleavage underlies proper spermatid differentiation.

In many animals, differentiating spermatids remodel their chromatins by replacing most histones with smaller basic proteins called protamines (*40*). Histone modifications including H4 hyperacetylation are associated with histone-to-protamine transition (*41*–*43*). Given that *nej* is regulated by CDS-piRNA pathway (Fig. 5), histone acetylation and associated transition events could be impaired by loss of *aub*. Consistent with previous studies, acetylated lysine (K) signals on H4 were detectable in spermatid nuclei (Fig. 6, A and B, and fig. S6) (*39*). During transition, reducing signals of H4 and the acetylated K5, K8, and K12 could overlap with accumulating protamine B (ProtB), but it lasts only transiently (Fig. 6B, top panels). Later, ProtB-positive and H4-negative nuclei further sharpened their structures and started to be individualized (fig. S6). By contrast, *aub* mutant testes maintained higher levels of H4K8Ac and H4K12Ac, known *nej*-sensitive modifications (*44*), compared to heterozygous control and transgene rescue conditions (Fig. 6C). In addition, *aub* mutant testes accumulated individualized nuclei that abnormally contain both ProtB and acetylated H4 (Fig. 6B, bottom panels). These results are in line with the notion that regulation of *nej* and other genes by CDS-piRNA pathway underlies proper chromatin remodeling and individualization during spermatid differentiation.

## DISCUSSION

Our bioinformatic, proteomic, and genetic approaches revealed a unique pathway in which hundreds of selected endogenous protein-coding genes contribute to noncoding piRNA biogenesis by providing precursors to Aub. Namely, CDS-piRNA production involves testis-specialized factors including Cyp40, Ago2, and binding si/miRNAs, but not Ago3 and Rhi, and hence, the pathway is distinguished from that of cluster/TE-piRNAs. How can two distinct piRNA biogenesis pathways cooperate in testicular germ cells? In testes, Ago3 is restricted in spermatogonia (*26*, *27*), and Rhi loses its expression in early spermatocyte differentiation (*28*). Hence, cluster/TE-piRNA biogenesis is supposed to be weakened as germ cells differentiate. On the other hand, Aub and Cyp40 are broadly expressed (*25*, *27*). We showed that CDS-piRNA production is inducible during spermatogonia-to-spermatocyte transition (fig. S1). Considering these, we propose that temporal restriction of cluster/TE-piRNA production in testes allows Aub to build up an alternate loading system for CDS-piRNAs (Fig. 6D). Now, it remains elusive what factor(s) essentially contribute to CDS-piRNA production. Nonetheless, we demonstrate that this pathway prefers transcripts targeted by Ago2 as precursors (Fig. 4). If transcript cleavage by Ago2 is necessary for piRNA, production awaits further investigation. Cyp40 can support CDS-piRNA production both directly through the interaction with Aub (Fig. 3) and indirectly through the modulation of Ago2 loading (*25*).

This study showed that piRNA production can be directed by both siRNAs and miRNAs binding to Ago2 in fly testicular germ line (Fig. 4). Now, it is uncertain how widespread si/miRNA-directed piRNA biogenesis is across animals. In many organisms including plants, fungi, and different animals, transcripts targeted by trigger sRNAs recruit RNA-dependent RNA polymerase (RdRp) enzymes, whose activities mediate silencing amplification through secondary siRNA biogenesis. In plants, mi/siRNA-directed transcript cleavage can recruit RdRp, and produced double-stranded RNAs are processed by RNase III enzymes into secondary siRNAs (*45*). In nematodes, transcripts targeted by primary piRNAs recruits RdRp. Synthesized short complementary fragments are directly incorporated into worm-specific Ago (WAGO) subfamily members as secondary, so-called 22G-siRNAs (*46*). *RdRp* genes can be found in the genomes of animals not restricted to nematodes. However, loss of *RdRp* has occurred frequently and independently during evolution, resulting in the sporadic absence of RdRp activities in organisms including *Drosophila* and mammals (*47*). The prevalence of si/miRNA-directed piRNA biogenesis, an RdRp-independent mechanism to amplify and diversify sRNAs, might have relevance to the absence of *RdRp* in genome.

An important aspect in secondary/responder sRNA biogenesis is to protect precursor transcripts from degradation and to promote their entry to processing pathways. In ping-pong amplification, a Tudor domain protein Krimper tethers piRNA-bound Aub and piRNA-free Ago3 in physical proximity to facilitate the efficient transfer of cleaved transcripts to Ago3 (*48*, *49*). In phased piRNA biogenesis, cleaved fragments are carried by RNA helicase Armitage to the processing center on mitochondrial outer membrane (*50*, *51*). On the other hand, in plant cells where secondary siRNAs accumulate, 3′ cleavage fragments and RdRp templates are stabilized by RISCs and associated factors (*52*, *53*). The above evidence lets us to hypothesize that *Drosophila* testicular germ cells may maintain mechanism(s) enabling the transfer of precursors to Aub for efficient CDS-piRNA production. Responsible factors might exist among those identified in the physical proximity of Cyp40 (Fig. 3) or could be identified in the interactome of Aub or Ago2 in future studies. It has been shown that, in plants, precursors can be stabilized by selected sRNAs, which are distinguished by their length of 22 nt but not the shorter isoforms (*52*, *54*–*57*). We mention that the major form of *CG4068* and *hpRNA1* siRNAs associated with CDS-piRNA production is exceptionally 22 nt in length (Fig. 4), which are distinct from the most abundant 21-nt ones in testicular pools. Perhaps not all siRNAs are capable of inducing CDS-piRNA production. In addition, our data do not exclude the possible involvement of Ago1. It is an open question whether any Ago- or sRNA-selective rule can be applied to define CDS-piRNA production triggers.

Why do testes produce CDS-piRNAs? Alhough we provided evidence that *nej* is one of the direct targets of CDS-piRNAs (Fig. 5), the global view of CDS-piRNA mode of actions during late spermatogenesis, when Aub exerts crucial functions, awaits further investigation. Murine pachytene piRNAs are expressed in neonatal testes accumulating spermatocytes and spermatids and critically regulate their differentiation (*18*, *58*, *59*). Extensive studies have presented compelling evidence supporting different, if not discrepant, conclusions regarding pachytene piRNA activities, including transposon silencing, genome integrity protection, endogenous mRNA degradation through deadenylation or cleavage, translational activation, and murine PIWI (MIWI) degradation through ubiquitination (*18*, *60*–*65*). Perhaps *Drosophila* CDS-piRNAs, having unique sequences like pachytene piRNAs, exhibit multiple activities. Notably, CDS-piRNAs are minor constituents of germline piRNAs in whole testis profiling; however, their abundance in late spermatocytes and spermatids might be underrepresented. Further dissection of CDS-piRNA activities could reveal the commonalities and differences of piRNA-mediated sperm formation between animals.

Hsp90 machinery controls the ligand binding processes of selected client proteins often through dynamic and transient interactions (*66*). These chaperone functions include loading/binding of sRNA precursors (ligands) to Ago family proteins (clients). Once loading occurs, Hsp90 changes its dimer conformation through adenosine 5′-triphosphate hydrolysis and dissociates from Ago complexes to complete RISC formation (*29*, *67*, *68*). Cochaperone Cyp40 functions with Hsp90 during RISC formation and transiently interacts with Ago proteins in cell lysate systems (*25*, *69*). However, the transient-thus-unstable binding nature makes cochaperone-client interactome difficult in tissue levels. By overcoming the above challenges with the proximity identification method, BioID/TurboID (*33*, *34*), this study identified otherwise undetected physical and functional links between Cyp40 and selected Ago members in testicular germ cells. BioID/TurboID combined with different genetic tools available in model organisms will further decipher selective (co)chaperone-client interactions within and beyond our scopes.

Last, the fact that endogenous genes can produce both proteins and regulatory RNAs raises a question of how sequences coding multiple functional factors arrange their genetic information through evolution. It would be interesting to investigate how protein-coding genes evolve in organisms producing CDS-piRNAs compared to those lacking the pathway and if there is any evidence supporting the selective pressures directing functionalization of CDS-piRNAs over proteins. An example might be *muc14A* that atypically uses antisense transcription for CDS-piRNA production, while the sense transcripts are below detectable levels as an exception of mucin family genes (*70*).

## MATERIALS AND METHODS

### Fly stocks and cultures

Fly stocks are reared at room temperature (RT) on a molasses/yeast medium [5% (w/v) dry yeast, 5% (w/v) corn flower, 2% (w/v) rice bran, 10% (w/v) glucose, 0.7% (w/v) agar, 0.2% (v/v) propionic acid, and 0.05% (v/v) *p*-hydroxy butyl benzoic acid]. The following stocks were used: *eGFP-Piwi* (*attP2*) (*71*), e*GFP-Aub/CyO* (DGRC, #118621) (*72*), *cyp40^KO^*/*TM3* (*25*), Df(3 L)BSC669/*TM6C* (BL, #26521) (deficiency for *cyp40*), *bam-Gal4/TM6*, *FLAG-HA-ago2* (*23*), *yellow white* (*y w*), *ago2^454^*/*TM3* (BL, #36512), *miR-316^KO^*/*TM3* (Kyoto, #116-356), *Df(3 L)ED4978/TM6c* (Kyoto, #150-442) (deficiency for *miR-316*), *Df(3 L)BSC558/TM6C* (BL, #25120) (deficiency for *ago2*), *dcr2^L811fsX^* (BL, #33053), *aub^HN2^*/*CyO* (BL, #8517), *aub^N11^*/*CyO* (*73*)*, ago3^t2^*/*TM6B* (BL, #28269)*, ago3^t3^*/*TM6B* (BL, #28270)*, rhi^02086^*/*CyO* (BL, #12226), *Df(2R)Exel7149/CyO* (BL, #7890) (deficiency for *rhi*), *protamine-B-eGFP*/*CyO* (DGRC, #109173), *nos-phiC31*; P{y[+t7.7] = CaryP}attP40 (BL, #25709), and *nos-phiC31*;; PBac{y[+]-attP-3B}VK00033 (BL, #32543).

### Plasmid construction and fly transformation

All the primers used for plasmid construction are listed in table S6. To generate UASp-*mTurbo-FLAG-cyp40* or UASp-*mTurbo-FLAG-cyp40^RKAA^*, *mTurbo* was amplified by pfusion PCR (NEB) using Ti833/Ti834 as primers and 3xHA-miniTurboNLS_pCDNA3 (Addgene, #107172) as template. *FLAG-cyp40* or *FLAG-cyp40^RKAA^* fragment was amplified by PCR using primers Ti835/Ti836 and UASp-*GFP-FLAG-cyp40* or UASp-*GFP-FLAG-cyp40^RKAA^* as template (*25*). Amplified *mTurbo* and *FLAG-cyp40* fragments were introduced into Xba I site of pUASp-K10-attB vector using the In-Fusion HD Cloning Kit (Takara). To generate UASp-*mTurbo-FLAG-cyp40^deltaTPR^,* a fragment containing *mTurbo-FLAG-cyp40^deltaTPR^* (primers: Ti835/484) was amplified by PCR using UASp-*mTurbo-FLAG-cyp40* as template and introduced into Xba I site of pUASp-K10-attB vector. The constructs were integrated into *attP40* site. To generate UASp-*mTurbo-FLAG-aub* (only used for rescue experiment in this study), *FLAG-aub* fragment (primers: Ti953/894) was amplified by PCR using *y w* cDNA as template. *mTurbo* and *FLAG-aub* fragments were introduced into Xba I site of pUASp-K10-attB vector. The construct was integrated into *attP-3B* site.

### Proximity-dependent biotin identification in testicular germ cells

mTurbo fusion proteins were expressed in germline-restricted manner under *bam* promoter activity using Gal4/UASp system. After eclosion, male progenies were reared at 25°C for 3 days in the modified molasses/yeast medium supplemented with 100 μM biotin (Nacalai). From 200 testes, biotinylated proteins were purified as described with some modifications (*34*). After exchanging the buffer with 100 μl of PI-lysis buffer [50 mM tris-HCl (pH 7.5), 500 mM NaCl, 2 mM EDTA, 2 mM dithiothreitol (DTT), 0.4% (w/v) SDS, and cOmplete Protease Inhibitor Cocktail Tablet (Roche)] testes were homogenized using Bioruptor (Diagenode) for 30 s (power H) for six times with 30-s intervals. Triton X-100 was added to sample mixtures at a final concentration of 2% (v/v), and homogenization was further performed for 30 s for three times with 30-s intervals. After centrifugation (20,000*g*, 10 min, 4°C), the supernatant was diluted by mixing with equal amount of 50 mM Tris-HCl (pH 7.5) solution and incubated with pre-equilibrated 15-μl slurry volume of Dynabeads MyOne Streptavidin C1 (Thermo Fisher Scientific) overnight at 4°C with gentle rotation. Next day, beads were washed twice with W1 buffer [50 mM tris-HCl (pH 7.5), 250 mM NaCl, 0.2% (w/v) SDS, 1 mM EDTA, and 1 mM DTT], twice with W2 buffer [50 mM Hepes-KOH (pH 7.4), 500 mM NaCl, 0.1% (w/v) deoxycholate (Nacalai), 1% (v/v) Triton X-100, and 1 mM EDTA], twice with W3 buffer [10 mM tris-HCl (pH 8.0), 250 mM LiCl, 0.5% (w/v) deoxycholate, 0.5% (v/v) NP-40 (Nacalai), and 1 mM EDTA], and twice with W4 buffer [50 mM tris-HCl (pH 7.5) and 50 mM NaCl]. Proteins bound to magnet beads were denatured by boiling at 95°C for 5 min in 2× protein loading buffer [4% (w/v) SDS, 200 mM DTT, 0.1% (v/v) bromophenol blue (BPB), and 20% (v/v) glycerol] saturated with biotin, resolved by SDS–polyacrylamide gel electrophoresis (SDS-PAGE) in 5 to 20% precast gel (ATTO). After electrophoresis, proteins were visualized by using the Silver Stain MS Kit (Wako). Proteins in gel particles were digested with trypsin and analyzed by liquid chromatography tandem MS (LC-MS/MS) using Q Exactive (Thermo Fisher Scientific) and UltiMate 3000 Nano LC (Thermo Fisher Scientific) in CoMiT Omics Center (Osaka University, Japan). The mass spectrum data were analyzed by Mascot v2.5.1 (Matrix Science). Proteins were identified with threshold of 1 peptide minimal. Pseudo value of 0.5 was added to all identified proteins, and then peptide count values were normalized by using total peptide counts in a certain condition.

### sRNA extraction from testes

RNA was extracted from six hundred testes of ≤3 days old using miRNeasy (QIAGEN). Less than 200-nt fragments were collected using miRelute column (QIAGEN). A total of 2 pmol of oligo DNA (5′-AGTCTTACAACCCTCAACCATATGTAGTCCAAGCAGCACT-3′) containing complementary sequence to 2*S* ribosomal RNA (rRNA) was added to 1 μg of RNA solution, and 2*S* rRNA hybridizing with oligo DNA was digested with RNase H (NEB). RNA mixture depleted of 2*S* rRNA was loaded onto 8 M urea-polyacrylamide gel (12%) and size-separated in parallel with RNA ladder (Dynamarker DM253, BioDynamics Laboratory Inc.) in 0.5× tris-borate EDTA buffer. Gel area within the range of 20 to 30 nt was excised, and contained RNAs were eluted in 300 mM NaOAc (pH 5.2) solution by dilution overnight at 4°C with gentle rotation. RNA fragments were precipitated in the presence of 80% (v/v) ethanol and glycogen (40 μg/ml) (Nacalai). RNA pellet was rinsed twice with 80% (v/v) ethanol and then resuspended in RNase-free water.

### Piwi or Aub immunoprecipitation and bound piRNA extraction

Five hundred testes of ≤3 days old were homogenized using plastic pestle (Biomasher, Nippi) in lysis buffer [30 mM Hepes-KOH (pH 7.4), 300 mM NaCl, 2 mM MgCl_2_, 2 mM DTT, 10% (v/v) glycerol, 0.5% (v/v) Triton X-100, and complete tablet]. After centrifugation (12,000*g*, 5 min, 4°C), to purify endogenous Aub proteins, the supernatant was incubated with anti-Aub antibodies (1:10) for 3 hours on ice then further incubated with the mixture of protein A Dynabeads and protein G Dynabeads (Thermo Fisher Scientific) overnight with gentle rotation. To purify GFP-Aub or GFP-Piwi, the supernatant was incubated with anti-GFP antibody-conjugated magnet beads [Medical and Biological Laboratories (MBL)] overnight with gentle rotation. The beads were washed five times with wash buffer [30 mM Hepes-KOH (pH 7.4), 800 mM NaCl, 2 mM MgCl_2_, 2 mM DTT, 10% (v/v) glycerol, 0.5% (v/v) Triton X-100, and complete tablet]. The bead-bound sRNAs were extracted with TRIzol LS reagent (Thermo Fisher Scientific) following the manufacturer’s protocol and precipitated in the presence of 50% (v/v) isopropanol and 20 μg of glycogen (Nacalai) overnight at −20°C. After centrifugation (20,000*g*, 20 min, 4°C), the pellet was rinsed twice with 80% (v/v) ethanol and then resuspended in RNase-free water.

### Deep-seq of sRNAs and data processing

The sRNA sequencing (sRNA-seq) libraries were constructed with NEB Next Multiplex Small RNA Library Prep Set for Illumina (NEB). After agarose gel electrophoresis, the fragments containing sRNAs were extracted and sequenced by Illumina HiSeq 2500. The library construction and deep-seq were performed in Research Institute for Microbial Disease (RIMD; Japan). Data processing was performed by using CLC Genomics Workbench (QIAGEN). Following the removal of 3′ adaptor sequence (5′-AGATCGGAAGAGCACACGTCT-3′), reads ranging from 23 to 29 nt were analyzed unless otherwise indicated. rRNA-, transfer RNA–, miRNA-, small nuclear RNA–, and small nucleolar RNA–mapping reads were considered as non-piRNAs and excluded (non-piRNA removal). Remaining reads were mapped without mismatch to the major piRNA clusters (*15*). Then, the remaining reads were mapped without mismatch to transposons (dros_na_transposons.embl; http://ftp.flybase.net/flybase/transposons/). Nonspecifically matching reads were mapped randomly. When comparing the abundance of piRNAs mapping to clusters or TEs between libraries in Fig. 4, mapping to clusters or TEs was performed independently (not sequentially) using reads remaining after non-piRNA removal. In these analyses, mismatch-allowing mapping (80% similarity) was also performed to analyze larger piRNA population. The results were consistent between with and without mismatch options. After the removal of cluster/TE-mappers, the remaining reads were mapped without mismatch to genome (BDGP6). Maximal number of hits for a read was 1 (This setting includes multimappers within a gene but excludes multimappers on multiple genes). Transcript per million (TPM) values for 23- to 29-nt RNAs were given by normalization with the sum of exon-mapping reads per kilobase (RPK) (same calculation method to transcriptome data). RPM values for 23- to 29-nt RNAs were given by normalization with total reads remaining after non-piRNA removal. To construct bedgraph, binary alignment map (bam) data exported from CLC were processed using bedtools (genomecov) with scaling factor calculated by reads remaining after non-piRNA removal. Within corresponding conditions, individual bedgraphs were merged (unionbedg) and then visualized in IGV (https://software.broadinstitute.org/software/igv/). To profile the lengths of gene mapping reads, total RNA bam data were first merged within in-house or public dataset (two or four data, samtools merge). In each dataset, reads mapping to a certain gene were extracted (samtools view), read length was obtained (samtools stats), the proportion was calculated within 18- to 29-nt RNAs, and mean values in two datasets were lastly obtained. In the case of analyzing gene groups, the mean of included genes was further calculated. To analyze nucleotide preference, six bam data were merged first and included reads were analyzed altogether for total RNAs, while two bam data (Aub-IP and GFP-Aub-IP in *cyp40^KO/6^* background) were merged for Aub-bound RNAs. From merged bam, the read sequence information was extracted (BBMap reformat.sh, .bam-to-.fa conversion). First to 23rd sequences of individual reads were further extracted (Seqkit), and the nucleotide probability was obtained for each nucleotide position using Geom_logo under R environment. To measure the density of Aub-bound piRNAs on 5′UTR, CDS, and 3′UTR, reads mapping to respective regions were counted (samtools view). Density was given by dividing read counts with length of 5′UTR, CDS, or 3′UTR. Relative density values were calculated, and the mean of Aub-IP and GFP-Aub-IP data was obtained. Values from 14 representative host genes were analyzed by box plot under R (ggplot2). To characterize piRNA strand orientation, mapping was performed in strand-specific manner using CLC (strand specific setting: forward or reverse), and the proportion of sense strand reads in total mapped reads was calculated. To identify 5′-to-5′ complementary 10-nt overlap pairs between siRNAs and piRNAs or between miRNAs and piRNAs, we developed a software complementary_analysis_v1_1 (https://doi.org/10.5061/dryad.79cnp5j19). The partners of hp-derived siRNAs and miRNA strands were searched within Aub-bound piRNAs mapping to endogenous gene exons. For this purpose, reads were merged between Aub-IP and GFP-Aub-IP (*cyp40^KO/6^* background) or between two replicates of Aub-IP (*y w* background). When >10 reads of piRNAs were paired with a certain si/miRNA (software version1_1 requesting 10 nt perfect complementarity without GU wobble), piRNA host genes were identified. piRNAs paired to miRNAs were analyzed only when the miRNA strand is detectable in testes and shows sorting preference to Ago2 (RPM Ago1 < Ago2) (*25*). To identify CDS-piRNAs that can target *nej*, CDS-piRNA reads derived from 324 host genes (RPM > 10) were collected from two libraries (Aub-IP and GFP-Aub-IP in *cyp40^KO/6^* background). Degradome reads mapping to *nej* were extracted from two libraries (PRJNA719671). Identical reads were grouped and then analyzed by complementary_analysis_v1_4 (https://doi.org/10.5061/dryad.79cnp5j19). piRNA groups containing >10 reads were reported. piRNA 11th nucleotide base pairing was requested for cleavage. Similar analysis was performed between the groups of CDS-piRNAs and *nej*-derived piRNAs. Phasing of CDS-piRNAs collected from two libraries (Aub-IP and GFP-Aub-IP in *cyp40^KO/6^* background) was analyzed by the published protocol (*74*). Analyzed sRNA data were summarized in table S1 (*75*–*79*).

### Transcriptome analysis

Two fly lines, *aub^HN2^/CyO; ago2^454^/TM6* and *aub^N11^/CyO; ago2^Df^/TM6*, were crossed to obtain male progenies of all genotypes analyzed by transcriptome in Fig. 5. From 150 to 200 testes of ≤3-day-old adults, total RNAs were extracted using TRIzol LS reagent (Thermo Fisher Scientific) following the manufacturer’s protocol and precipitated in the presence of 50% (v/v) isopropanol and 20 μg of glycogen (Nacalai) overnight at −20°C. After centrifugation (20,000*g*, 20 min, 4°C), the pellet was rinsed twice with 80% (v/v) ethanol and then resuspended in RNase-free water. After treating with deoxyribonuclease (DNase) I (NEB), RNA mixture was added with equal volume of phenol:chloroform:isoamyl alcohol (25:24:1, pH 5.2, Nacalai), vortexed, and centrifuged (20,000*g*, 3 min), and the supernatant was collected. After repeating this step, RNA in the supernatant was precipitated in the presence of 0.3 M NaOAc (pH 5.2) and 70% (v/v) ethanol overnight at −20°C. After centrifugation (20,000*g*, 20 min, 4°C), the pellet was rinsed twice with 80% (v/v) ethanol, resuspended in RNase-free water, and stored at −80°C until shipment. Library construction and deep-seq were performed by Rhelixa (Japan). Polyadenylated RNA selection was performed with NEBNext Poly(A) mRNA Magnetic Isolation Module. Libraries were constructed with the NEBNext Ultra II Directional RNA Library Prep Kit and sequenced by using NovaSeq 6000 (Illumina). The reads were processed and analyzed with CLC genomics workbench (QIAGEN). Paired reads were mapped in the default setting on *D. melanogaster* genome (BDGP6). The maximal number of hits for a read was 10. Using read counts on exons from biological duplicate samples, the differential expression analysis was conducted with EdgeR under R environment. Differentially expressed genes were selected with the threshold of FDR < 0.0001 (intersect), and venn diagram was drawn under R. Genes identified in individual categories (*aub* mutant, *ago2* mutant, or dKO) were listed in table S5. Gene Ontology analyses were performed in WebGestalt (www.webgestalt.org/). bedgraph was made with bedtools using bam data exported from CLC. Scaling factor was given by total counts of exon mapped reads. Bedgraph was visualized in IGV. For transcriptome data used in Fig. 1, RNA (>200 nt) was extracted from 400 testes of ≤3 day-old-flies using miRNeasy Mini column (QIAGEN). For the first replicate, extracted RNA was treated with DNase I (NEB) and aRibo-Zero rRNA removal kit (Illumina). After purifying RNA, the libraries were constructed with the TruSeq Standard mRNA Library Prep Kit (Illumina) and sequenced with HiSeq 3000 (Illumina) in RIMD (Japan). For the second replicate, polyadenylated RNAs were collected after DNase I treatment. Polyadenylated RNA enrichment, library construction (NEBNext Ultra Directional RNA Library Prep Kit for Illumina), and sequencing with HiSeq X (Illumina) were performed by AnnoRoad Co. Ltd. (China).

### Immunoblotting

Protein samples were denatured by boiling at 95°C for 3 min in protein-loading buffer [2% (w/v) SDS, 100 mM DTT, 0.05% (v/v) BPB, and 10% (v/v) glycerol], resolved by SDS-PAGE, and transferred to 0.2-μm polyvinylidene difluoride membrane (Wako) using the semi-dry system (Trans-blot Turbo, Bio-Rad). The membrane was blocked in 4% (w/v) skim milk (Nacalai) in 1× phosphate-buffered saline (PBS) supplemented with 0.1% (v/v) Tween 20 and further incubated with the following antibodies: anti-Aub antibody (1:500; guinea pig) (*80*), anti-GFP antibody (1:2000; Clontech, rabbit), anti-Ago1 antibody (1:1000; Abcam, Ab5070, rabbit), anti-Ago2 antibody (1:100; guinea pig) (*25*), anti-Piwi (1:10; mouse, P4D2), anti-FLAG M2-peroxidase [horseradish peroxidase (HRP)] (1:5000; Sigma-Aldrich, #A8592), anti-H3K18Ac (1:2000; Active Motif, #39756), anti-H3K27Ac (1:2000; Active Motif, #39136), anti-H4K8Ac (1:2000; Active Motif, #61104), anti-H4K12Ac (1:2000; Active Motif, #39928), anti-guinea pig immunoglobulins-HRP (1:1000; Dako), anti-rabbit immunoglobulin G (IgG)–HRP (1:3000; Bio-Rad), anti-mouse IgG-HRP (1:3000; Bio-Rad). The chemiluminescent signals were obtained by using Chemi-Lumi One (Nacalai) and detected by Chemidoc MP Imaging system (Bio-Rad). The images were processed by using Pixelmator or Fiji. The immunoblot signals were quantified by using Fiji.

### Histochemistry and image acquisition

Testes were dissected from adult males in 1× PBS buffer supplemented with 0.4% (w/v) bovine serum albumin (BSA; Wako) and fixed in 5.3% (v/v) paraformaldehyde (Nacalai) in 0.67× PBS buffer for 10 min. To observe DNA and ICs, testes were incubated with 1 μM 4′,6-diamidino-2-phenylindole and phalloidin Rhodamine X conjugated (1:1000; Wako) in PBX buffer [1× PBS containing 0.2% (v/v) Triton X-100]. To observe ProtB-GFP, the emission signal of GFP was acquired. For immunostaining, fixed testes were washed with PBX and incubated with PBX containing 2% (w/v) BSA for 30 min for blocking. The primary antibody incubation was performed overnight at 4°C, and testes were washed with PBX at 25°C for 1 hour. The secondary antibody incubation was then performed at 25°C for 2 hours, and then testes were washed with PBX at 25°C for 1 hour. The following antibodies were used: anti-Vasa antibody (1:5000; guinea pig), anti-guinea pig IgG–Alexa Fluor 555 (1:500; Molecular probes), anti-H3K18Ac (1:200; Active Motif, #39756), anti-H3K27Ac (1:200; Active Motif, #39136), anti-H4K5Ac (1:200; Active Motif, #39170), anti-H4K8Ac (1:200; Active Motif, #61104), anti-H4K12Ac (1:200; Active Motif, #39928), anti-H4 (1:200; Active Motif, #61300), and anti-rabbit IgG-Alexa Fluor 555 (1:500; Molecular probes). Images were acquired using confocal microscope LSM 900 (Zeiss). Images were processing by using Zen (Zeiss) and Pixelmator.

### Quantivative reverse transcription PCR

RNA was extracted from ~20 testes of ≤3-day-old flies for each condition using TRIzol LS reagent (Thermo Fisher Scientific) following the manufacturer’s protocol. Using DNase I (NEB)–treated RNA, cDNA was synthesized with 2.5 μM oligo(dT) adaptor using SuperScript III reverse transcriptase (Thermo Fisher Scientific). Quantivative reverse transcription PCR (qPCR) reaction was performed using KAPA SYBR FAST qPCR Master Mix (Roche) and gene-specific primers (table S6) in QuantStudio 5 Real-Time PCR system (ABI).

### Male fertility test

Single male was mated with six *y w* virgin females for the first 3 days and with another six *y w* virgin females the following 2 days at 25°C. The total number of hatched eggs was counted.

## Notes

View/request a protocol for this paper from *Bio-protocol*.
