# Supplementary material for: miRNA/siRNA-directed pathway to produce noncoding piRNAs from endogenous protein-coding regions ensures Drosophila spermatogenesis
Source: Sci Adv. 2023 Jul 19;9(29):eadh0397. doi: 10.1126/sciadv.adh0397 (PMC10355832; doi:10.1126/sciadv.adh0397)
Supplement: Supplementary file 1 — Figs. S1 to S6 Legends for tables S1 to S6 [file sciadv.adh0397_sm.pdf]

Science Advances
AAAS

Supplementary Materials for

miRNA/siRNA-directed Pathway to Produce Non-coding piRNAs from Endogenous Protein-coding Regions Ensures Drosophila Spermatogenesis

Taichiro Iki *et al.*

Corresponding author: Taichiro Iki, ikit@fbs.osaka-u.ac.jp.

Sci. Adv. **9**, eadh0397 (2023)
DOI: 10.1126/sciadv.adh0397

The PDF file includes:

Figs. S1 to S6
Legends for tables S1 to S6

Other Supplementary Material for this manuscript includes the following:

Table S1 to S6

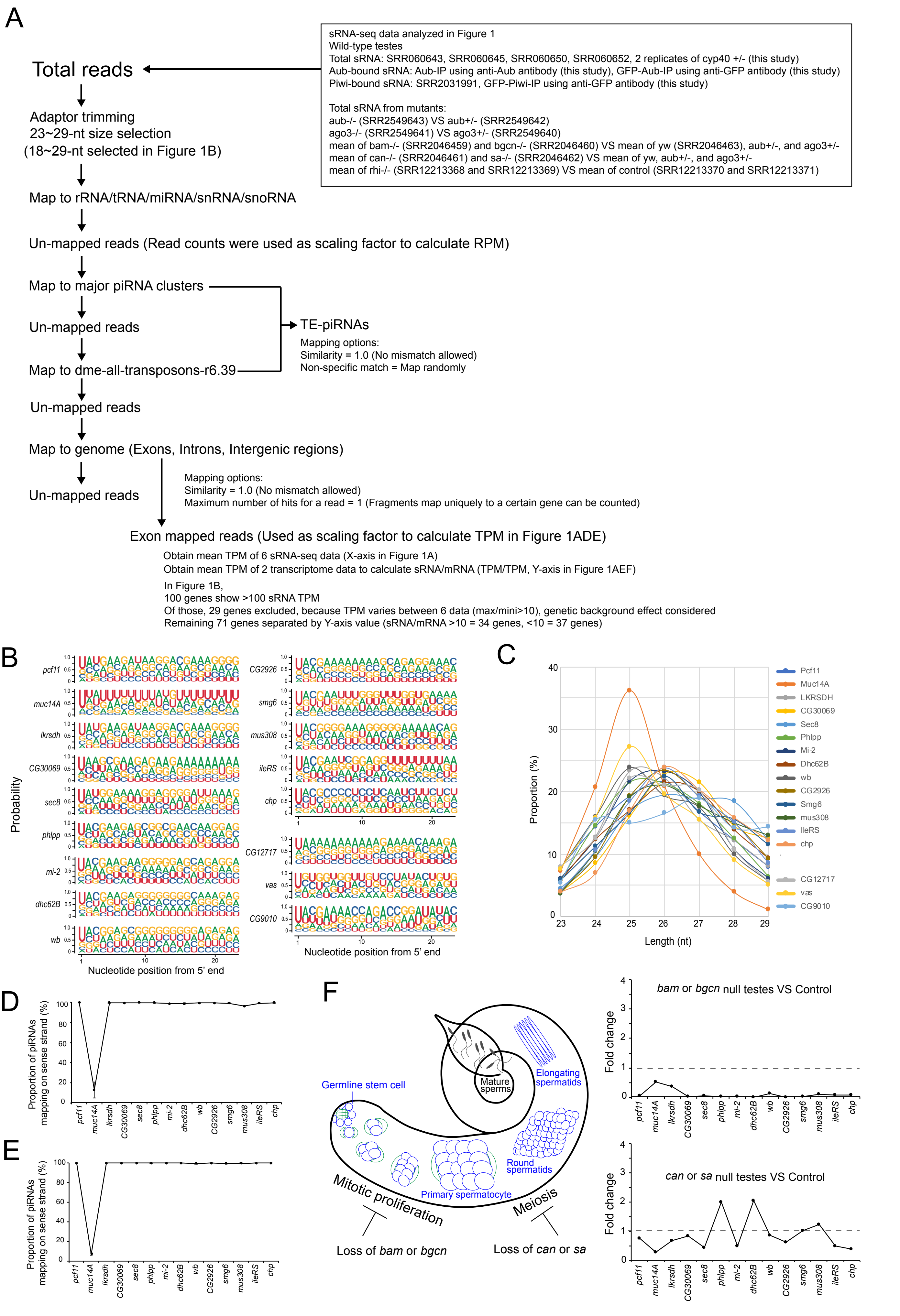
A
Total reads
Adaptor trimming
23~29-nt size selection
(18~29-nt selected in Figure 1B)
Map to rRNA/tRNA/miRNA/snRNA/snoRNA
Un-mapped reads (Read counts were used as scaling factor to calculate RPM)
Map to major piRNA clusters
Un-mapped reads
Map to dme-all-transposons-r6.39
Un-mapped reads
Map to genome (Exons, Introns, Intergenic regions)
Un-mapped reads
TE-piRNAs
Mapping options:
Similarity = 1.0 (No mismatch allowed)
Non-specific match = Map randomly
Mapping options:
Similarity = 1.0 (No mismatch allowed)
Maximum number of hits for a read = 1 (Fragments map uniquely to a certain gene can be counted)
Exon mapped reads (Used as scaling factor to calculate TPM in Figure 1ADE)
Obtain mean TPM of 6 sRNA-seq data (X-axis in Figure 1A)
Obtain mean TPM of 2 transcriptome data to calculate sRNA/mRNA (TPM/TPM, Y-axis in Figure 1AEF)
In Figure 1B,
100 genes show >100 sRNA TPM
Of those, 29 genes excluded, because TPM varies between 6 data (max/mini>10), genetic background effect considered
Remaining 71 genes separated by Y-axis value (sRNA/mRNA >10 = 34 genes, <10 = 37 genes)
sRNA-seq data analyzed in Figure 1
Wild-type testes
Total sRNA: SRR060643, SRR060645, SRR060650, SRR060652, 2 replicates of cyp40 +/- (this study)
Aub-bound sRNA: Aub-IP using anti-Aub antibody (this study), GFP-Aub-IP using anti-GFP antibody (this study)
Piwi-bound sRNA: SRR2031991, GFP-Piwi-IP using anti-GFP antibody (this study)
Total sRNA from mutants:
aub-/- (SRR2549643) VS aub+/- (SRR2549642)
ago3-/- (SRR2549641) VS ago3+/- (SRR2549640)
mean of bam-/- (SRR2046459) and bgcn-/- (SRR2046460) VS mean of yw (SRR2046463), aub+/-, and ago3+/-
mean of can-/- (SRR2046461) and sa-/- (SRR2046462) VS mean of yw, aub+/-, and ago3+/-
mean of rhi-/- (SRR12213368 and SRR12213369) VS mean of control (SRR12213370 and SRR12213371)
B
Probability
pcf11
muc14A
lkrsdh
CG30069
sec8
phlpp
mi-2
dhc62B
wb
CG2926
smg6
mus308
ileRS
chp
CG12717
vas
CG9010
Nucleotide position from 5' end
C
Proportion (%)
Length (nt)
Pcf11
Muc14A
LKRSDH
CG30069
Sec8
Phlpp
Mi-2
Dhc62B
wb
CG2926
Smg6
mus308
IleRS
chp
CG12717
vas
CG9010
D
Proportion of piRNAs mapping on sense strand (%)
E
Proportion of piRNAs mapping on sense strand (%)
F
Germline stem cell
Mature sperms
Elongating spermatids
Round spermatids
Primary spermatocyte
Mitotic proliferation
Meiosis
Loss of bam or bgcn
Loss of can or sa
bam or bgcn null testes VS Control
can or sa null testes VS Control
Fold change

Figure S1. Testicular small RNA mapping to D. melanogaster gene exons

(A) Scheme for deep-sequencing data processing. (B, C) Nucleotide probability and size distribution of 23~29-nt RNAs in Aub-bound fraction. Reads in 2 data (Aub-IP and GFP-Aub-IP) were merged and analyzed. (D, E) Sense/antisense strand bias of 23~29-nt RNAs mapping to indicated genes. In panel D, in-house and public data for testicular total small RNAs were analyzed separately, and mean values of sense strand proportion were obtained. In panel E, 2 data for Aub-bound RNAs (Aub-IP and GFP-Aub-IP) were analyzed separately, and mean values of sense strand proportion were obtained. (F) Effect of loss of germline differentiation factors on the expression of 23~29-nt RNAs in testes. *bag of marbles (bam)* or *benign gonial cell neoplasm (bgcn)* mutants accumulate early stage spermatogonial cells, while *cannonball (can)* or *spermatocyte arrest (sa)* mutants accumulate primary spermatocytes.

A

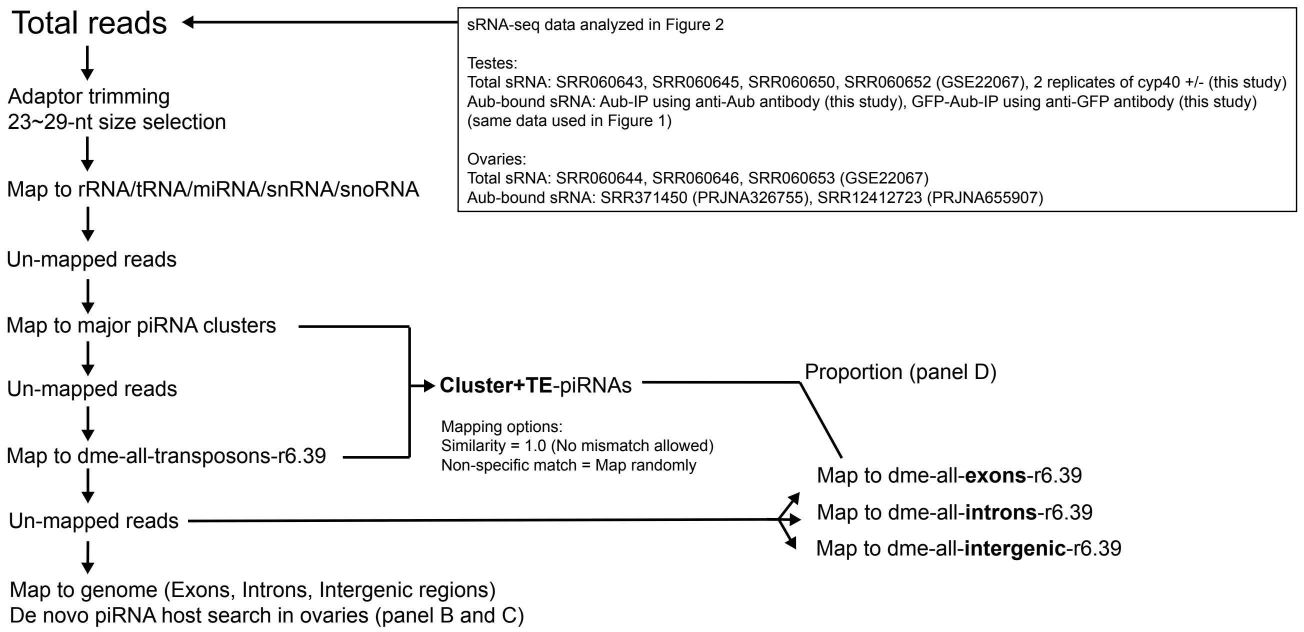

B

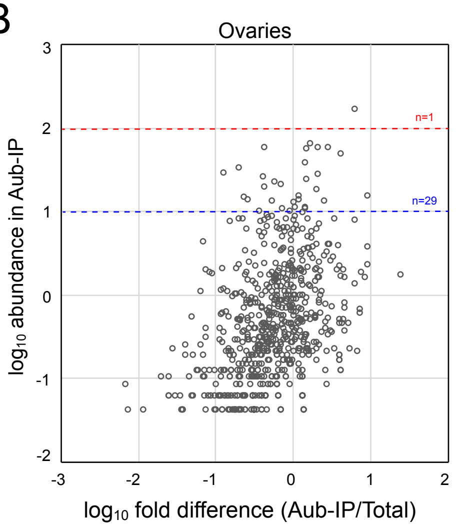

1st filter: 23~29-nt sRNA/mRNA (TPM/TPM) >10
Testis: 2449 genes
Ovaries: 910 genes

2nd filter (loose): Aub-bound piRNA (RPM) >10
Testis: 324 genes
Ovary: 29 genes

2nd filter (strict): Aub-bound piRNA (RPM) >100
Testis: 17 genes
Ovary: 1 gene

C

Genes	Mean RPM in ovarian Aub-IP
RpL10Ab	162.3244626
RpL3	61.36694079
Stt3A	56.61918361
rl	56.05558953
CG17514	55.86731562
Maf1	54.68528491
CG17493	47.91498149
Muc14A	36.34839476
Nipped-A	32.28901014
CG32000/anne	27.48714437
CG30069	26.58828949
CG17715	24.93818175
Pcf11	23.49794121
CG40160	21.46545766
RpS2	20.07012238
sta	18.62288824
UQCR-11	17.37135061
CG11023	14.72711646
Sox102F	14.67000212
Cp15	14.19609711
CG41099	13.44944461
CG3704	13.15681991
l(3)76BDm	12.99376016
RpL24	12.81971897
Atf6	12.27502
CG3812	10.8319572
toy	10.59736691
l(2)k0120	10.39559687
RpS15	10.35786869

These genes were identified as piRNA-hosting genes in testes.

D

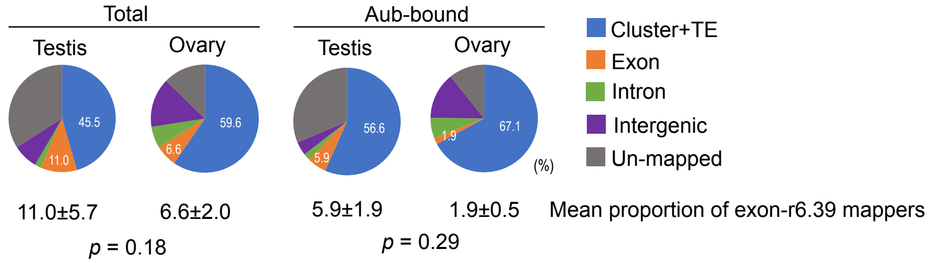

Figure S2. Comparative analysis of ovarian and testicular small RNAs

(A) Scheme for deep-sequencing data processing for comparing testes and ovaries. (B) Search for endogenous protein-coding genes accumulating 23~29-nt RNAs in ovaries. As in analyses on testicular RNAs, 910 genes were first extracted by the abundance of 23~29-nt fragments relative to mRNAs (TPM/TPM). Of those 910 genes, only one gene (RpL10Ab) produced >100 RPM potential piRNAs, and only 29 genes produced >10 RPM potential piRNAs in Aub-bound fraction. (C) List of 29 genes producing Aub-interacting potential piRNAs in ovaries. Mean RPM was given by 2 Aub-IP data (SRR371540 and SRR12412723). Genes in red color were identified in testes as hosts of piRNAs. The differences of piRNA abundance between testes and ovaries can be seen in Figure 2B. (D) Pie charts show the proportions of 23~29-nt RNAs mapping to indicated genomic contents.

A

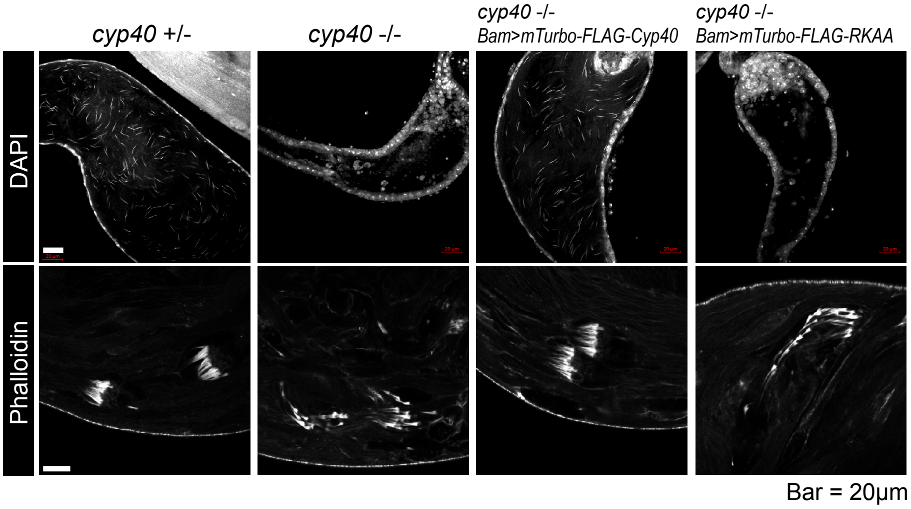

B

Name	Enrichment	p (<0.01)	Coverage (%)					
		mTurbo:	Cyp40	Cyp40	RKAA	ΔTPR	GFP	GFP
**Gene silencing**								
Dcr2	12.94	2.61169E-06	7	7	0	0	0	0
Ago2	11.37	0.000122319	10	8	0	0	0	0
Aub	8.81	0.000370819	21	21	1	0	4	0
Qin/Kumo	5.91	0.001117021	4	4	1	0	0	0
fs(1)Yb	5.29	0.00219343	5	7	0	0	0	1
**Nuclear**								
CG9899	23.50	4.38273E-05	51	45	4	0	1	1
Mle	12.52	0.00015714	20	24	1	0	3	1
Sbf	10.88	0.000216629	6	8	1	0	0	0
Mus308/Pol θ	3.02	0.008841278	1	1	0	0	0	0
**Microtubule**								
CG17687	39.72	0.000260524	19	21	0	0	0	0
CG14838/Mmm	19.74	5.19148E-06	22	19	1	0	0	0
Klp10A	6.06	0.000151872	7	6	0	0	0	0
**Mitochondrion**								
Nrd1	22.67	7.46998E-07	20	18	0	0	1	0
CG3107	9.90	3.74038E-06	7	8	0	0	0	0
Ogdh	3.81	5.77854E-05	2	2	0	0	0	0
**tRNA metabolism**								
MetRS	4.92	0.005576408	40	21	2	3	6	3
ValRS	3.81	5.77854E-05	3	2	0	0	0	0
**mRNA metabolism**								
Bgcn	16.88	0.004128041	8	12	0	0	0	0
Irp-1a	12.84	0.001757869	19	11	0	0	0	0
Upf1	4.59	0.003436041	2	3	0	0	0	0
Gld2	1.79	0.007425165	10	8	3	2	5	4
**Other**								
Ubr1	5.98	0.004688514	2	4	0	0	1	0
CG14411	5.33	1.62642E-05	6	6	0	0	0	0
Synj	3.02	0.008841278	2	1	0	0	0	0
Naa15-16	3.02	0.008841278	3	1	0	0	0	0

C

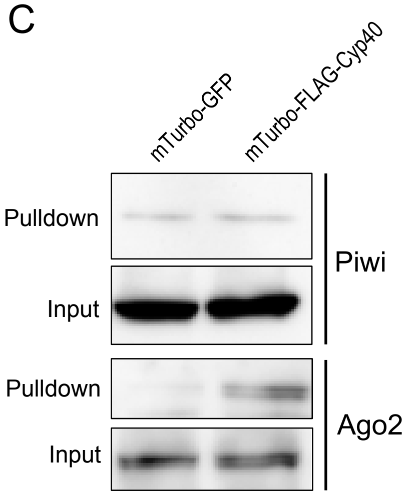

Figure S3. Cyp40 client screening in testicular germ cells using TurboID

(A) Defective spermatogenesis in testes lacking *cyp40* (-/-; *cyp40*$^{KO/Df}$), and the rescue by *mTurbo-FLAG-cyp40* transgene expressed under germline-specific *bam* promoter activity. Nuclei of mature sperms stored in seminal vesicles were observed with DAPI, and individualization complexes (ICs) in elongating spermatids were observed with phalloidin. *cyp40*^{RKAA} (*mTurbo-FLAG-RKAA*) is a non-functional variant of *cyp40*. Heterozygous sibling (+/-) serves as wild-type control. (B) List of proteins identified in the physical proximity of Cyp40. The values for enrichment and statistic difference (*p*) correspond to those shown in Figure 3C. Table also contains peptide coverage (%) of identified proteins. Identified proteins were classified into groups by their known or predicted functions. (C) Immunoblotting of Piwi and Ago2 present in testes (Input) and purified with streptavidin (Pulldown).

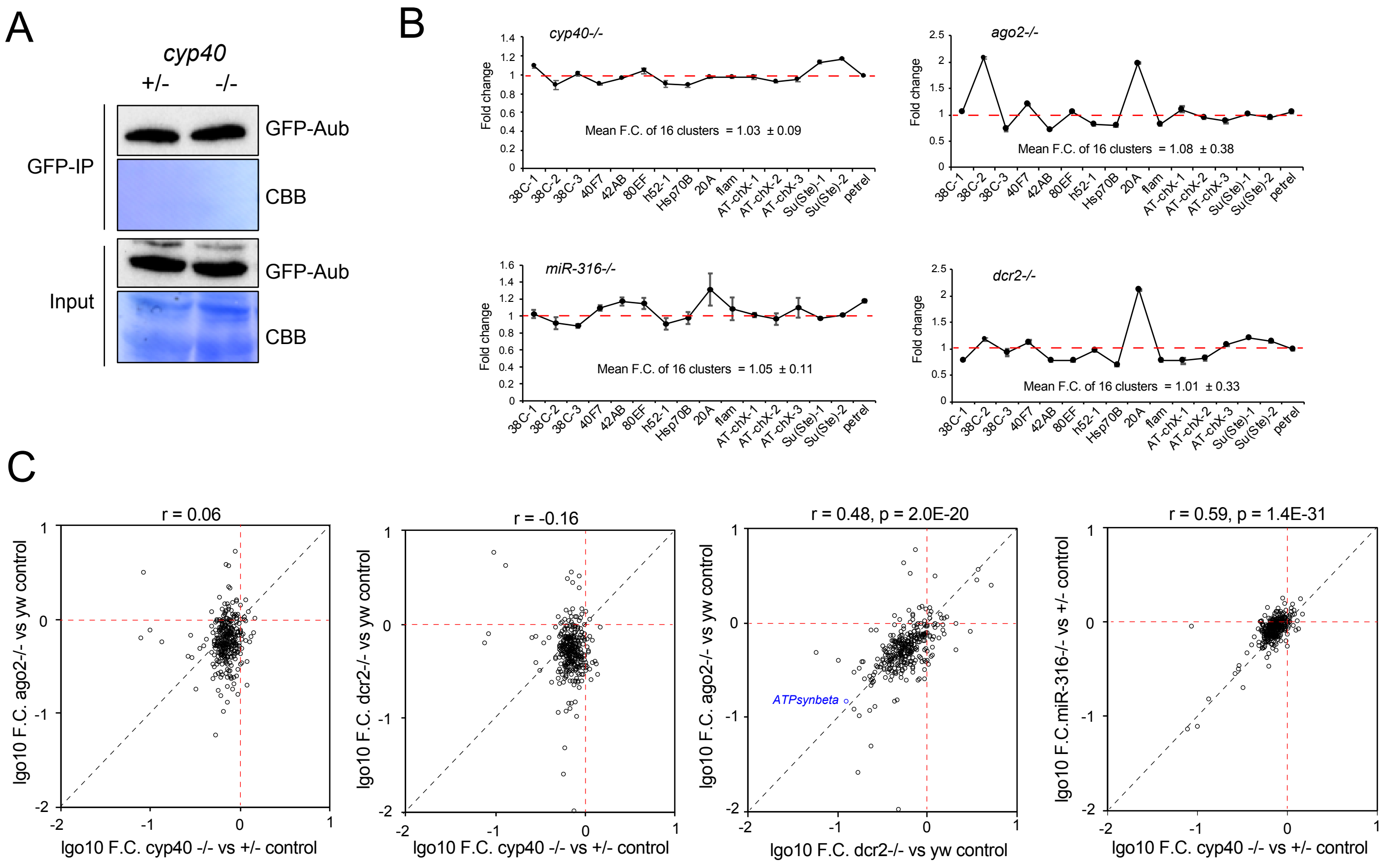

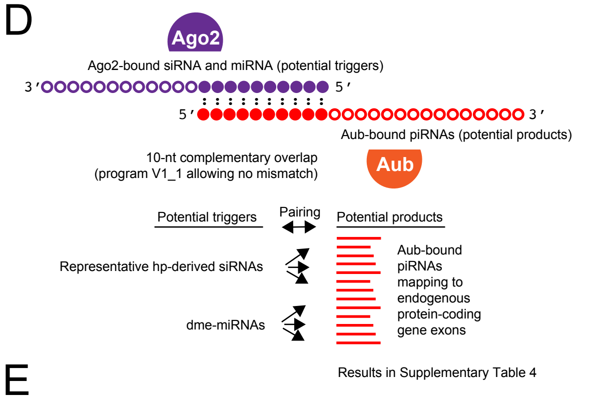

E

Tandem repeat finder - Input: *muc14A* coding-region antisense strand (48672nt)

Indices	Period Size	Copy Number	Consensus Size	Percent Matches
3227--11061	285	27.3	285	94
11061--15266	285	14.7	285	95
20350--25641	291	18.2	290	95
25642--45132	291	66.5	291	90

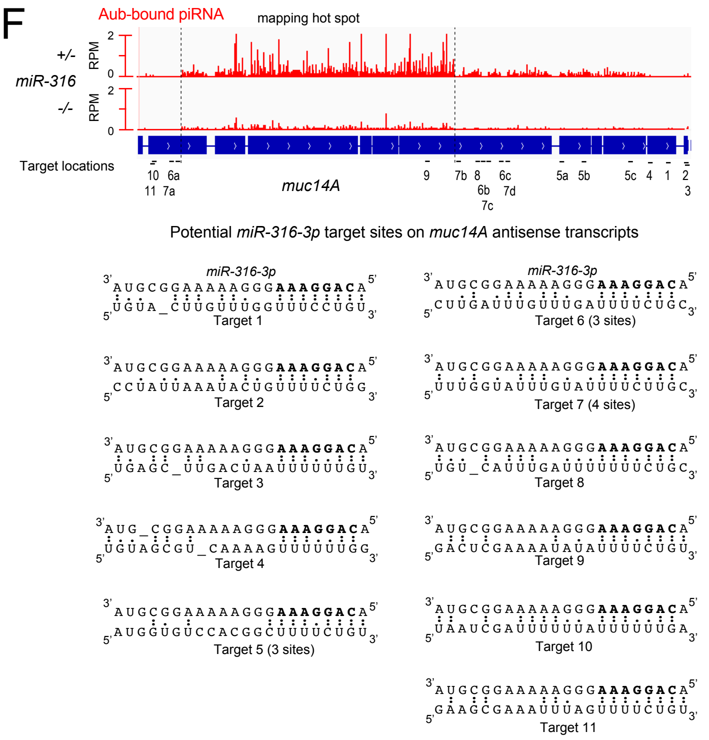

G

Gene	phasing z-score	read strand
muc14a	3.771185	-
chp	2.958918	+
phlpp	2.50385	+
sec8	2.340151	+
pcf11	2.175257	+
smg6	1.757222	+
dhc62B	1.549734	+
lkrsdh	1.22056	+
CG30069	1.169575	+
CG2926	1.032143	+
IleRS	0.7143963	+
mi-2	0.631252	+
wb	0.519125	+
mus308	-0.08640092	+
ATPsynbeta	0.3991698	+

Figure S4. Molecular requirement for CDS-piRNA biogenesis

(A) Immunoblotting of GFP-Aub proteins present in testes of heterozygous (+/-) and homozygous (-/-) of *cyp40* (Input) and those purified with anti-GFP antibodies for deep-sequencing (GFP-IP). CBB staining serves as protein loading control. (B) Effect of loss of *cyp40*, *ago2*, *dcr2*, or *miR-316* on the accumulation of cluster-mapping piRNA inside Aub-RISCs. Mean ± s.d. of 2 data set was shown. (C) Correlation analyses. F.C. of piRNA abundance (mutant/control) was compared between two conditions. Correlation coefficient (r) and *p* value are shown. (D) Screening of 5'-to-5' 10-nt complementary overlap. piRNAs paired with siRNAs or miRNAs were searched within Aub-bound exon mappers. (E) Repeat sequence analysis on *muc14A* protein coding-region. Tandem repeat finder (https://tandem.bu.edu/trf/home) was used with default parameters. (F) piRNA origins and predicted *miR-316-3p* target sites on *muc14A*. Seed sequence (2-8th) of *miR-316-3p* is shown in bold letters. Target sites were identified if they contain complementary sequences to the seed (allowing GU wobble pairs). (G) Phasing analysis on CDS-piRNAs. Phasing z scores were calculated for individual piRNA-hosting genes.

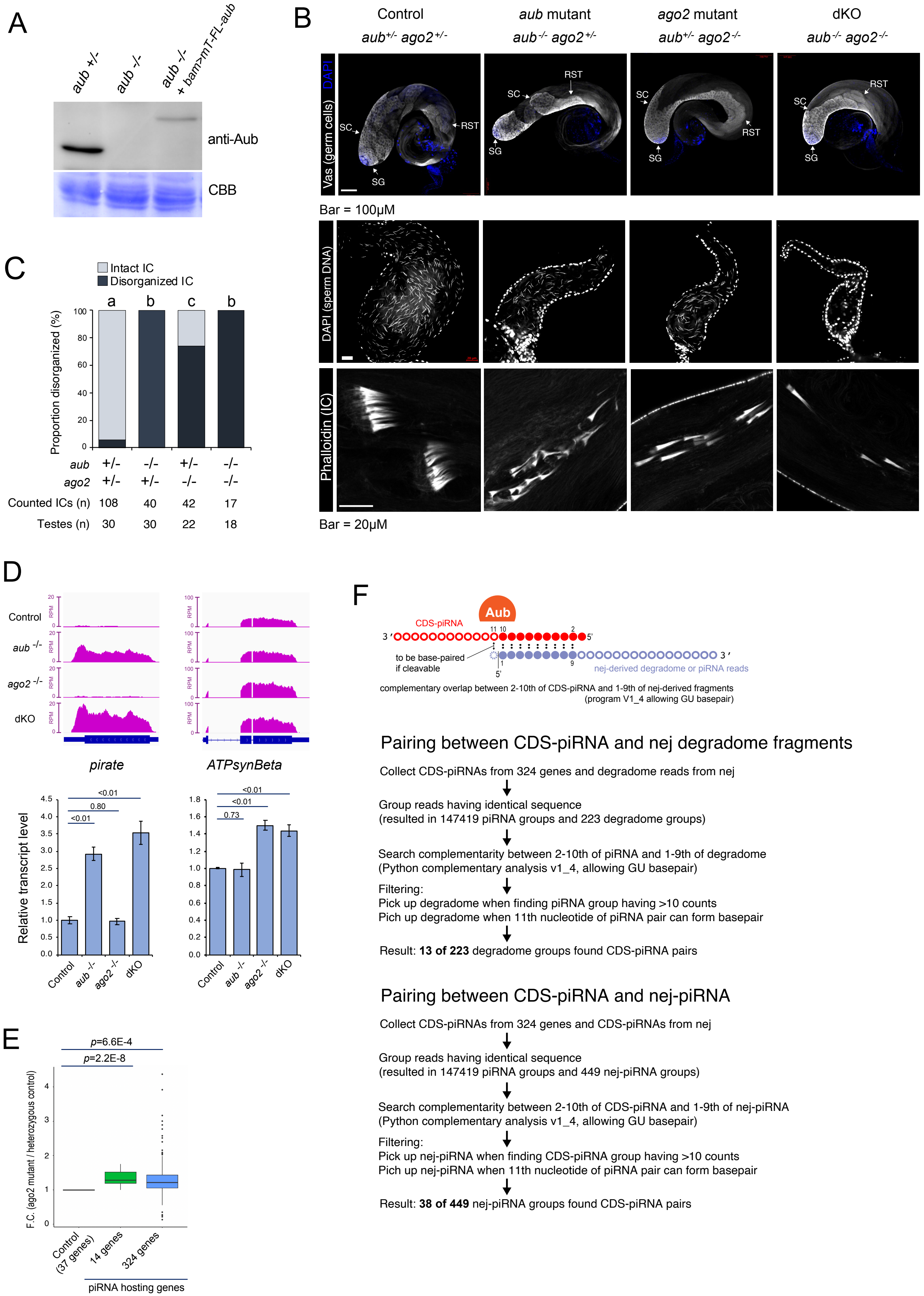
A
aub +/-
aub -/-
aub -/- + bam>mT-FL-aub
anti-Aub
CBB
B
Control
$aub^{+/-}$ $ago2^{+/-}$
aub mutant
$aub^{-/-}$ $ago2^{+/-}$
ago2 mutant
$aub^{+/-}$ $ago2^{-/-}$
dKO
$aub^{-/-}$ $ago2^{-/-}$
Vas (germ cells) DAPI
SC
SG
RST
Bar = 100μM
DAPI (sperm DNA)
Phalloidin (IC)
Bar = 20μM
C
Intact IC
Disorganized IC
a b c b
Proportion disorganized (%)
aub +/- -/- +/- -/-
ago2 +/- +/- -/- -/-
Counted ICs (n) 108 40 42 17
Testes (n) 30 30 22 18
D
Control
$aub^{-/-}$
$ago2^{-/-}$
dKO
RPM
pirate
ATPsynBeta
Relative transcript level
<0.01
0.80
<0.01
<0.01
<0.01
0.73
Control
aub -/-
ago2 -/-
dKO
E
p=6.6E-4
p=2.2E-8
F.C. (ago2 mutant / heterozygous control)
Control (37 genes)
14 genes
324 genes
piRNA hosting genes
F
Aub
CDS-piRNA
3′ 5′
11 10 2
to be base-paired if cleavable
1 9
5′ 3′
nej-derived degradome or piRNA reads
complementary overlap between 2-10th of CDS-piRNA and 1-9th of nej-derived fragments
(program V1_4 allowing GU basepair)
Pairing between CDS-piRNA and nej degradome fragments
Collect CDS-piRNAs from 324 genes and degradome reads from nej
Group reads having identical sequence
(resulted in 147419 piRNA groups and 223 degradome groups)
Search complementarity between 2-10th of piRNA and 1-9th of degradome
(Python complementary analysis v1_4, allowing GU basepair)
Filtering:
Pick up degradome when finding piRNA group having >10 counts
Pich up degradome when 11th nucleotide of piRNA pair can form basepair
Result: 13 of 223 degradome groups found CDS-piRNA pairs
Pairing between CDS-piRNA and nej-piRNA
Collect CDS-piRNAs from 324 genes and CDS-piRNAs from nej
Group reads having identical sequence
(resulted in 147419 piRNA groups and 449 nej-piRNA groups)
Search complementarity between 2-10th of CDS-piRNA and 1-9th of nej-piRNA
(Python complementary analysis v1_4, allowing GU basepair)
Filtering:
Pick up nej-piRNA when finding CDS-piRNA group having >10 counts
Pich up nej-piRNA when 11th nucleotide of piRNA pair can form basepair
Result: 38 of 449 nej-piRNA groups found CDS-piRNA pairs

Figure S5. Functions of Aub, Ago2, and CDS-piRNAs in late spermatogenesis

(A) Immunoblotting of Aub proteins in testes of heterozygous control (+/-), or of *aub*$^{N11/HN2}$ null mutants (-/-) in the absence or presence of *mTurbo-FLAG-aub* transgene expression. CBB staining serves as protein loading control. (B) Phenotypes of testes lacking *aub*, *ago2*, or both. Male progenies of *aub*$^{N11/HN2}$ mutant (*aub*$^{-/-}$ *ago2*$^{+/-}$), *ago2*$^{454/Df}$ mutant (*aub*$^{+/-}$ *ago2*$^{-/-}$), double knock-out (dKO) (*aub*$^{-/-}$ *ago2*$^{-/-}$), or heterozygous sibling control (*aub*$^{+/-}$ *ago2*$^{+/-}$) were obtained from a single mating. Top panels show DAPI and Vas (germline marker) signals in the whole testes. SG; spermatogonia, SC; spermatocytes, RST; round spermatids. Lower two panels show nuclei of sperms stored in seminal vesicles (DAPI), and ICs formed by spermatids (Phalloidin). (C) Disorganized IC counting. Number of analyzed testes and counted ICs were indicated. Differential characters indicate statistically significant difference (p<0.01) in Tukey's test. (D) Transcriptome (bedgraph) and qPCR measurement (bar graph) data on *pira* and *ATPsynbeta*. Mean ± s.d. of biological triplicate data was shown. *P* values given by two-tailed unpaired *t*-test were indicated. (E) Transcript fold changes in ago2 mutants were compared between CDS-piRNA hosting genes (n=14 or 324) and piRNA-poor control genes (n=37 shown in Figure 1AB). *p*; two-tailed unpaired *t*-test. (F) Analysis of 5'-to-5' 10nt overlap pairs between CDS-piRNAs and nej-derived degradome fragments or piRNAs.

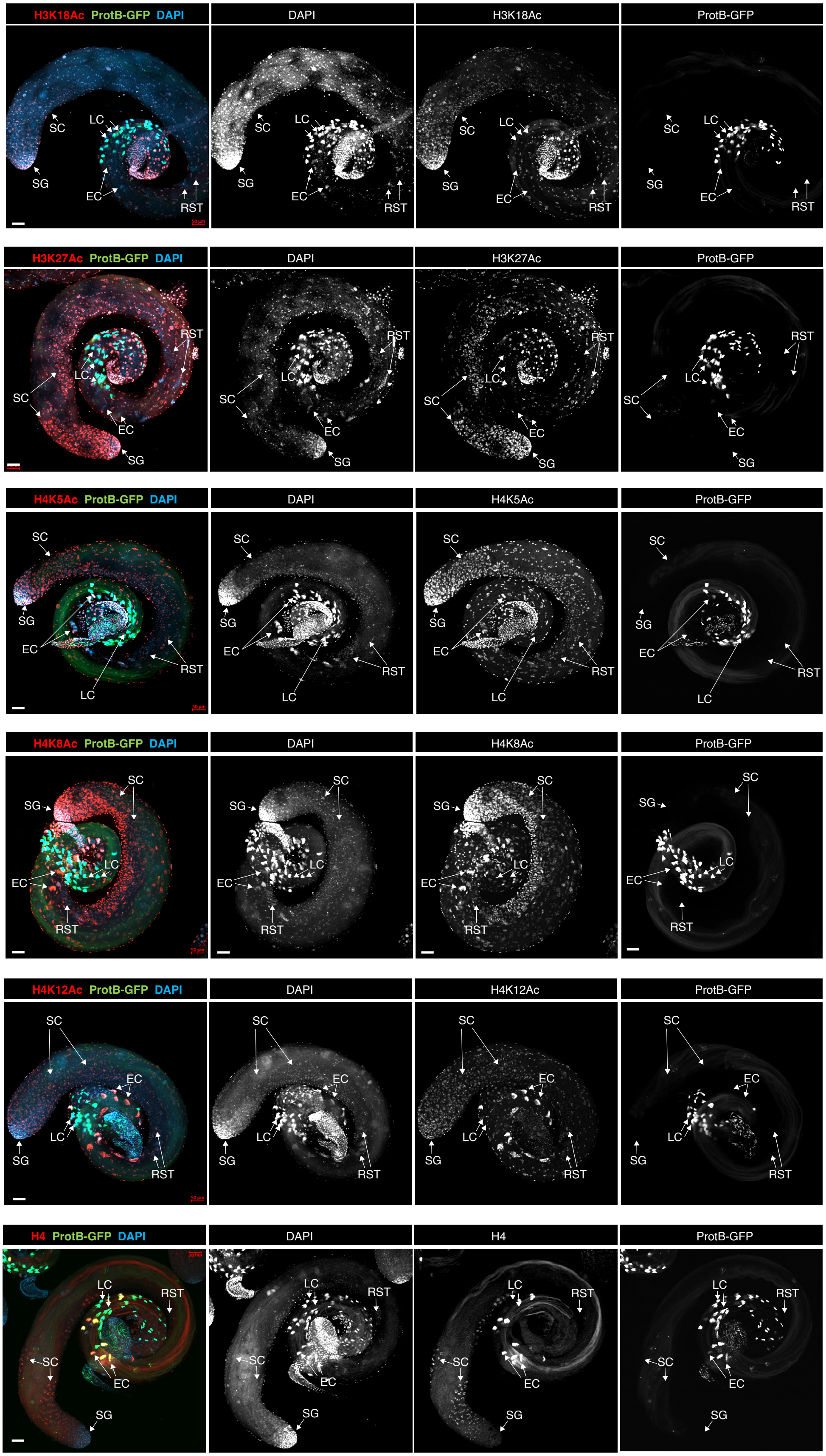

H3K18Ac ProtB-GFP DAPI
DAPI
H3K18Ac
ProtB-GFP
SC
LC
SG
EC
RST
H3K27Ac ProtB-GFP DAPI
H3K27Ac
H4K5Ac ProtB-GFP DAPI
H4K5Ac
H4K8Ac ProtB-GFP DAPI
H4K8Ac
H4K12Ac ProtB-GFP DAPI
H4K12Ac
H4 ProtB-GFP DAPI
H4

Figure S6. Histone H3 H4 acetylation signals in testes

Histone H3 H4 acetylation patterns in testes expressing ProtB-GFP. SG; spermatogonia, SC; spermatocytes, RST; round spermatids, ES; elongating spermatids, EC; early canoe stage spermatids, LC; late canoe stage spermatids. The observed acetylation signals were summarized in Figure 6A.

Captions for the supplementary tables.

Table S1. Deeq-sequencing data analyzed in this study.

Table S2. 5' nucleotide enrichment and size profile of unique mappers on 324 genes.

Table S3. List of 324 genes and the normalized counts of mapping reads.

Table S4. Ten nucleotide overlap pairs between Aub-bound CDS-piRNA and Ago2-bound si/miRNA.

Table S5. Mis-regulated genes in aub and ago2 mutants.

Table S6. Oligonucleotides used in this study.
